# Multiphysics Modeling of Photoelectrochemical Devices for Simultaneous Solar-Driven Biomass Reforming and Hydrogen Production

**DOI:** 10.1021/acs.energyfuels.5c01590

**Published:** 2025-06-18

**Authors:** Andrés F. Pérez Torres, Heejung Kong, Senapati Sri Krishnamurti, Feng Liang, Sixto Gimenéz, Roel van de Krol, Marco Favaro

**Affiliations:** † Institute for Solar Fuels, 28340 Helmholtz-Zentrum Berlin für Materialien und Energie GmbH, Hahn-Meitner-Platz 1, 14109 Berlin, Germany; ‡ Institut für Chemie, Technische Universität Berlin, Straße des 17. Juni 124, 10623 Berlin, Germany; § Institute of Advanced Materials, Universitat Jaume I, Avinguda de Vicent Sos Baynat, s/n, 12006 Castellón de la Plana, Spain

## Abstract

Biomass reforming, including glycerol and 5-hydroxymethylfurfural oxidation, converts renewable biomass-derived molecules into value-added chemicals and fuels. This process is crucial for sustainable energy and chemical production, offering a carbon-neutral alternative to fossil-based feedstocks. Integrating biomass oxidation with photoelectrochemistry enables solar-driven reactions, reducing external electrical input and improving energy efficiency. Photoelectrochemical cells selectively oxidize biomass-derived compounds at the photoanode while generating hydrogen or other reduction products at the cathode, creating a synergistic system for sustainable fuel and chemical production. Electrolyte transport properties significantly impact membraneless PEC device performance. This study systematically investigates flow behavior, crossover effects, and device operation using a 0.5 M glycerol solution as the anolyte. Despite its similar density and viscosity to water, the glycerol solution exhibits density-driven instabilities, leading to electrolyte mixing when paired with a pure water catholyte. Simulations reveal that using the same glycerol solution in both compartments prevents crossover and enhances stability. A single-bridge design optimized to minimize *iR* drop while maintaining separation reduced voltage losses by 47% compared to a double-bridge configuration. At flow rates ≥60 mL/min, product crossover remains negligible, supporting the feasibility of membraneless PEC designs for glycerol oxidation. These findings contribute to scaling up PEC systems for sustainable hydrogen and high-value-added chemical production, emphasizing the potential of modular, high-efficiency solar-driven biomass reforming.

## Graphical abstract

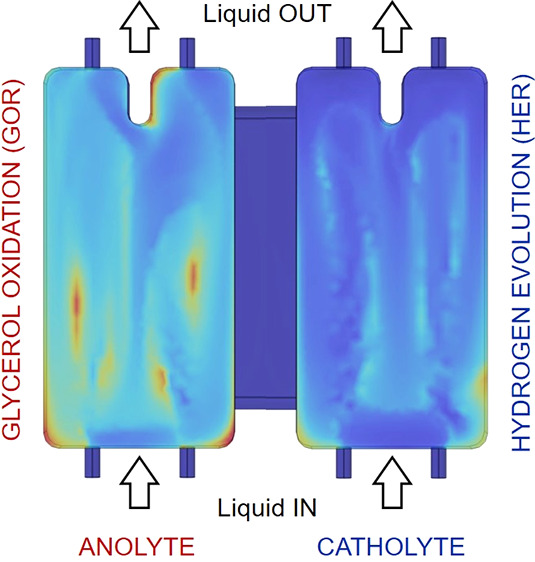

## 1 Introduction

Photoelectrochemical (PEC) water splitting is a technology aimed at producing green hydrogen by directly utilizing sunlight and water as the sole energy sources and feedstocks. However, the projected cost of hydrogen (10–20 $/kg) is significantly higher than that of hydrogen produced via methane reforming (1–2 $/kg), primarily due to challenges related to scalability and efficiency.^1^ Systems employing earth-abundant materials often exhibit low solar-to-hydrogen efficiencies, whereas higher efficiencies—up to 19%—have been achieved only by using costly III–V semiconductors^2,3^ or other materials designated as ‘critical raw materials’ (CRMs) by the European Commission.^4^ Photovoltaics coupled with electrolysis (PV/electrolysis) is a more established approach, however requiring separate investments in PV panels, electrolyzers, and the necessary electronic control and conversion devices (i.e., DC-DC converters). In contrast, integrated PEC devices can combine solar harvesting and electrolysis in a single, integrated unit. This offers the benefits of efficient thermal integration and lower current densities, thereby relaxing the high activity requirements imposed by the usual catalysts used in PV/electrolysis.^5^

Nonetheless, making PEC water splitting economically viable at a scale large enough to impact society remains a major challenge, motivating the search for alternative strategies. One possibility is to reduce the required energy input—specifically, by replacing the thermodynamically demanding oxygen evolution reaction (OER) with the oxidation of biomass-derived waste products, while still reducing protons to hydrogen at the cathode. Not only this requires less potential than water splitting: biomass oxidation products have, in general, much higher economic value than oxygen. Since biomass-derived molecules are more complex than the simple water molecule, controlling the selectivity of the oxidation reaction becomes challenging. Here, the PEC route may offer advantages, since it is possible to tune the surface energetics of the semiconducting photoanode (e.g., by doping or via the engineering of surface states), thereby improving the reaction selectivity beyond what is typically feasible with PV/electrolysis.^5^

The OHPERA project, one of nine selected by the European Innovation Council (EIC) for the European Hydrogen Portfolio in the period 2022–2026, aims to address these challenges through two novel approaches. First, OHPERA employs lead-free halide perovskite nanocrystals (PNCs) as highly efficient photoactive materials, known for their excellent light-harvesting capabilities.^6–9^ Here, PNCs serve as both the photoanode and the photocathode, supporting earth-abundant cocatalysts. Second, the OHPERA device replaces the OER with the glycerol oxidation reaction (GOR) at the anode. Glycerol, a major byproduct of the biodiesel industry, is upgraded through oxidation into higher-value products such as dihydroxyacetone (DHA), glyceraldehyde (GLAD), and glycolaldehyde (GCAD).^10,11^ These oxidation reactions also coproduce protons, which serve as input for the hydrogen evolution reaction (HER) that takes place at the cathode. The glycerol oxidation products offer significantly greater economic value than either oxygen or glycerol,^12–15^ as for example DHA can cost up to 702 €/g, GLAD 124 €/g, and GLAC 3290 €/g in contrast to glycerol (0.23 €/g).^15^ Furthermore, the oxidation reaction of glycerol to DHA has a formal potential (*E* ^0^) of approximately 0.18 V versus the reversible hydrogen electrode (V_RHE_) at 25 °C, which is notably lower than that of the OER (1.23 V_RHE_).^13^ This advantage may lead to higher solar-to-hydrogen efficiency due to the reduced energy input required for device operation;^1,8^ however, given the limited availability of biomass-derived wastes compared to water, these oxidation processes (including GOR) should be viewed as a complementary approach rather than a replacement for water splitting, offering an alternative pathway where biomass-derived feedstocks are accessible. For instance, PEC plants could be operating downstream of industrial sites that generate biomass-derived waste, utilizing these feedstocks directly at the production point to generate chemicals and fuels, thereby reducing both transportation costs and associated emissions.

For the design of the photoelectrochemical GOR/HER device, the OHPERA consortium has selected a side-by-side configuration, in which the photoanode and photocathode are placed next to each other and expose an area of 10 cm^2^ each to the light and the liquid electrolyte. Figure 1 presents a schematic of the PEC device design incorporating this configuration. The technical specifications of the device are described in the Supporting Information (Supplementary Note 1). This approach offers several advantages over the stacked configuration, in which the photoanode and photocathode face each other with an electrolyte and a membrane in between. In a stacked configuration, the two photoelectrodes should have different absorption ranges (i.e., bandgaps) to optimize light utilization.^16^ In contrast, the side-by-side configuration eliminates this requirement, as optimal sunlight exposure for both electrodes is ensured by positioning the photoanode and photocathode adjacent to each other^9,10^ (see Figure 1a,b). This point is particularly important for the OHPERA project, as the PNC-based photoabsorbers used in the device are intrinsic semiconductors. Their *p*-type (photocathode) or *n*-type (photoanode) character is determined solely by the nature of the back contact–either a hole-transport layer (photocathode) or an electron-transport layer (photoanode). Consequently, both photoabsorbers share the same bandgap and overall light absorption properties, which precludes the use of a stacked device. Additionally, the side-by-side geometry can ease fabrication constraints related to bubble management, especially when the photoabsorbers are back-side illuminated.^17,18^ Finally, implementing a mirror geometry to direct light to both sides of a stacked architecture would add a level of complexity that would hinder the future device’s scalability. Additionally, the side-by-side design prevents optical losses caused by separators such as ion exchange membranes (IEMs) or bipolar membranes (BPMs). This configuration also eliminates potential efficiency losses due to light attenuation or misalignment, which are more common in stacked configurations.^19^ However, a major limitation of the side-by-side device configuration is its less efficient use of incident light. Since each photon effectively illuminates only one of the two electrodes, the device requires twice the active area to absorb the same amount of sunlight as a stacked tandem design. As a result, the solar-to-hydrogen (or fuel) efficiency is inherently lower, potentially reducing overall performance by half compared to an optimized tandem architecture. On the other hand, a side-by-side configuration could facilitate maintenance and replacement of individual components, as each part is more accessible, thereby reducing long-term operational costs. In addition, this configuration could potentially lead to an easier scaling up, as it avoids the intricate layering required in stacked configurations.^2^ Hence, a trade-off must be carefully considered when evaluating the scalability and practical implementation of side-by-side designs, particularly in applications where maximizing light absorption and land use efficiency is critical.

**Figure 1:**
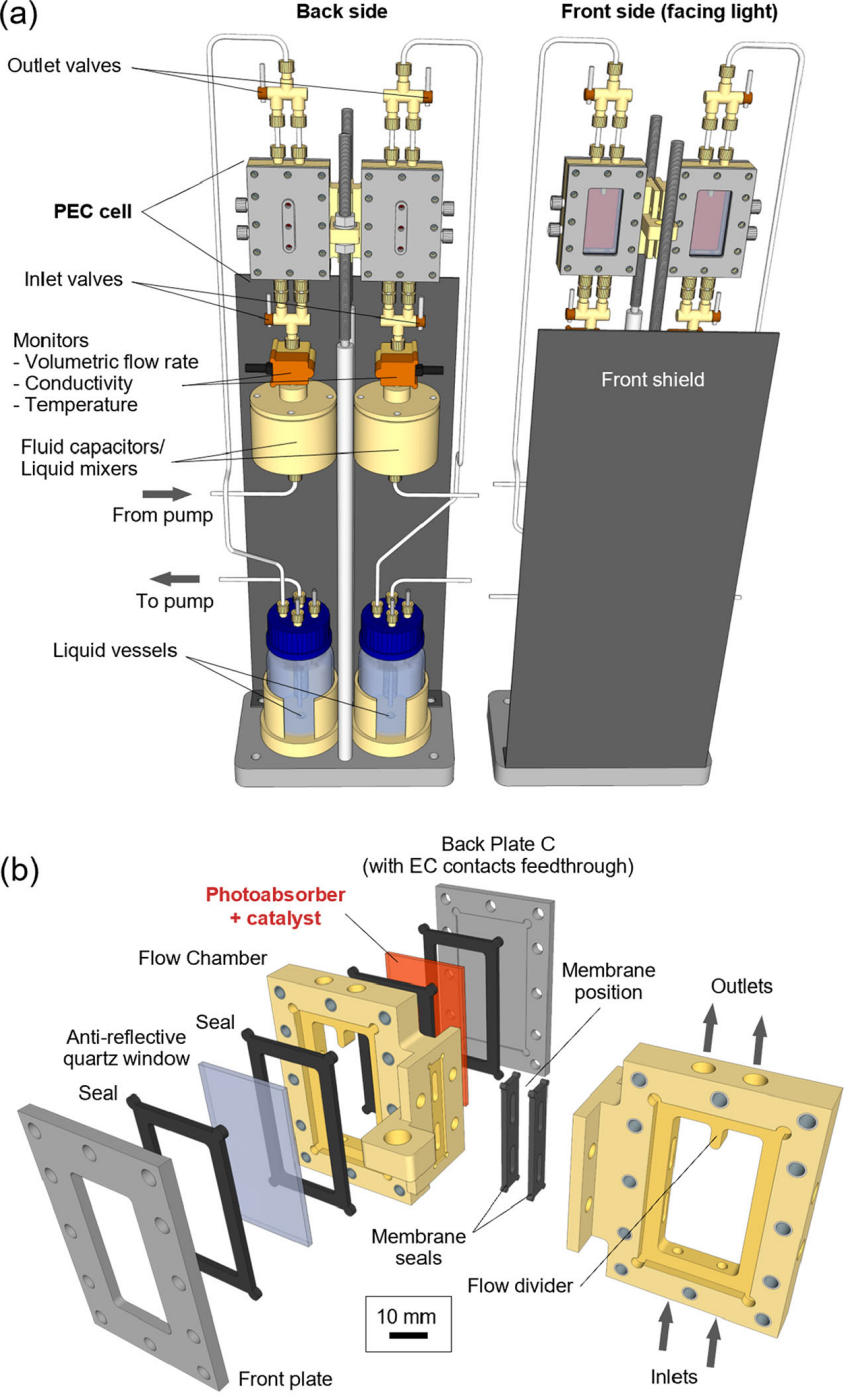
(a) Schematic illustrating the overall design of the side-by-side device selected by the OHPERA consortium; (b) exploded view of the photoelectrochemical (PEC) cell. The symmetric anolyte and catholyte compartments are connected by bridges where membranes can potentially be placed, if required. Each flow chamber has two inlets at the bottom and two outlets at the top (2 mm diameter), where also a fin dividing the liquid flow is present. Glycerol oxidation occurs on the photoanode while hydrogen evolution takes place at the photocathode. Each photoelectrode exposes an area of 10 cm^2^ to the light and the liquid electrolyte. Note that the device is designed to operate with or without a separating membrane for increased flexibility.

An important aspect of the PEC device depicted in Figure 1 is its flexibility regarding membrane usage. While membranes prevent mixing of products and enable separate pH conditions, they also come with significant drawbacks: increased complexity, cell voltage losses, potential durability issues, and high manufacturing costs.^16,20,21^ We estimated the normalized cost of the Nafion membrane per unit of power output for a PEC device operating at 10 mA/cm^2^, a target operational value for the OHPERA project. To do so, we interpolated data from an electrolyzer cost model developed by Thunder Said Energy, which takes into account a Nafion unitary cost of 2000 $/m^2^ (around 1760 €/m^2^ in May 2025).^22,23^ As previously reported by Strathmann et al.,^24^ the data show that the power-normalized cost of the membrane drastically increases with decreasing current density, given the associated decrease of the H_2_ production yield (hence limiting the economic return of operating the electrolyzer). At a current density of 10 mA/cm^2^, the extrapolated power-normalized cost of the membrane amounts to approximately 1900 €/kW, which is prohibitively expensive (for comparison, the power-normalized cost of membranes in an electrolyzer operating at 1 A/cm^2^ amounts to 100 $/kW,^23^ around 88 €/kW). This underscores the potential benefit of developing membraneless photoelectrolyzers for PEC technology, where the current densities are much lower than those usually reached in PV/electrolysis. This benefit is particularly important for medium-to-low scales, where the amount of evolved H_2_ does not represent a safety concern as well.^16^

Beyond economic considerations, the environmental impact of commonly used membranes is even more critical. Many of these membranes rely on per- and polyfluoroalkyl substances (PFAS), or “forever chemicals,” which persist in the environment and pose serious health risks.^25,26^ In response to these concerns, the European Union has taken strong actions^27–29^ and is moving toward even more stringent regulations that will ban the use of PFAS-based materials in the coming years.^30^ This policy shift reflects a broader commitment to reducing the environmental footprint of energy processes and promoting sustainable alternatives. Consequently, developing effective, sustainable alternatives to membranes has become a priority for electrolyzer research and industry.

In this work, we design a membraneless PEC device with a side-by-side configuration for simultaneous glycerol valorization and hydrogen production, guided by multiphysics modeling. By coupling fluid dynamics and mass transport simulations, we pinpoint strategies to minimize crossover between GOR products and hydrogen. Through this integrated modeling approach, we demonstrate how careful tuning of geometrical and operational parameters can maintain product purity without relying on PFAS-based membranes. Although this approach requires operating at a single pH and potentially contending with gas crossover, it eliminates concerns about membrane degradation, PFAS-related hazards, and additional manufacturing costs—ultimately aligning with our goal of creating a more economical and environmentally friendly PEC system.

## 2 Methods

### 2.1 Geometry

For the simulations two different geometries and dimensionalities were considered. A 2D geometry was used to study the effect of fluid properties on flow behavior, and a three-dimensional (3D) model was used to study the effect of flow rate and bridge size on crossover and *iR* drop (Figure 2).

**Figure 2:**
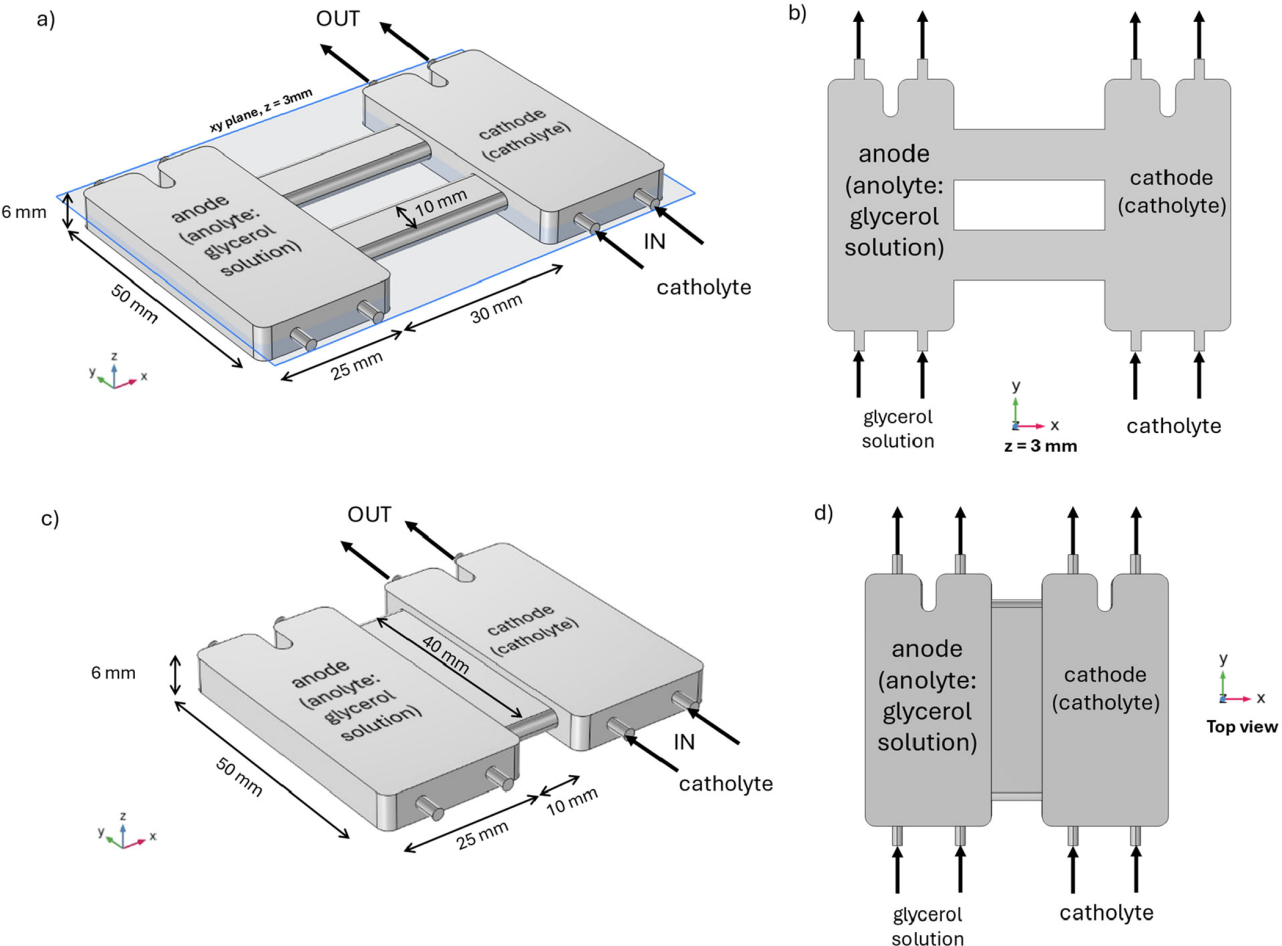
(a) Three-dimensional (3D) isometric view of the flow chambers of the PEC device with two-bridge configuration. “IN” and “OUT” denote the flow inlets and outlets, respectively. (b) Two-dimensional (2D) geometry of the photoelectrochemical (PEC) device. It consists of the cross section of the 3D geometry with the *xy* plane at *z* = 3 mm. This is the geometry employed to study the effect of fluid properties on flow behavior. (c) 3D isometric view of the flow chambers of the PEC device with one-bridge configuration. (d) Top view of the one-bridge configuration.

The simpler 2D model was used to study the effect of the fluid properties on flow profile, as it requires less computational power and allows performing more computationally intensive calculations. Within this set of simulations, two different types of fluid properties (viscosity and density) were considered. In the first case, we assumed that the concentration of glycerol is low enough (0.5 M) to consider that the change in the viscosity and density is negligible, and thus we used the properties of water. In the second case we evaluated this assumption and performed simulations that take into account how the density and viscosity of the mixture depend on the concentration of glycerol. The transport properties of the mixture (density and viscosity) were taken from a model and data reported in our previous work,^13^ selecting a temperature of 25 °C and a glycerol concentration range between 0 and 5M. To decrease computational costs and ease convergence, the data was fitted to a polynomial function and included in the COMSOL model (Figure S1). For the 2D simulations, glycerol solution is fed to the left compartment (anolyte), while pure water is fed to the right compartment (catholyte) (Figure 2b). In the 3D simulations, glycerol solution is fed to both compartments. For the 2D model, after a mesh independence study (Figure S2), a mesh with a fine size was chosen, corresponding to 11,356 elements (Figure S3). For these simulations, we employed the flow rates and glycerol concentrations displayed in Table 1.

**Table 1:** Molar Concentrations and Volumetric Flow Rates Used to Investigate the Dependence of Density and Viscosity on Glycerol Concentration

molar concentration (M)	flow rate (mL/min)
0.5	20
1	50
3	100
5	150
	200

The next step of the simulations was to use a 3D model, which resembles the geometry of the flow chamber and gives a more realistic behavior of the fluid flow within the cell. In this case, two different geometries were employed: one featuring two bridges connecting the electrochemical cells, and another with a single, shorter bridge of larger cross-sectional area linking the cells (Figure 2a,c, respectively). Results from the 2D simulations were employed for selecting the fluid properties and flow rate. After a mesh refinement study a mesh with fine size was chosen (Figure S4), with flow rates of 10–100 mL/min in steps of 10 mL/min.

### 2.2 Computational Fluid Dynamics (CFD)

CFD simulations were performed using the Laminar Flow interface of the CFD module in COMSOL Multiphysics 6.2 under steady-state conditions. The Laminar Flow Interface solves the continuity equation (eq 1) for low Reynolds (*Re*) numbers, along with the Navier–Stokes equation (eq 2):

∇·(ρu)=0 1

$$\rho (u\cdot \nabla u)=-\nabla p+\nabla \cdot \left(\mu (\nabla u+(\nabla u{)}^{T})-\frac{2}{3}\mu (\nabla \cdot u)I\right)+F$$ 2

where ρ is the density of the fluid (kg/m^3^), ***u*** the velocity (m/s), *p* the pressure (Pa), μ the dynamic viscosity of the fluid (Pa · s), and ***F*** the external force term (e.g., gravity, ρ · *g* · *h*). Both equations are solved in conjunction. The continuity equation (eq 1) enforces the conservation of mass, while the Navier–Stokes equation (eq 2) enforces the conservation of momentum. At the solid/liquid interface, Dirichlet boundary conditions are applied, setting ***u*** to zero (i.e., the no-slip condition).

The inlet flow rates for the catholyte and anolyte were specified as a fully developed flow, indicating that the fluid enters the cell in a laminar regime with a parabolic velocity profile in the inlet tubes. The flow rates for both the anolyte and catholyte compartments are set to the same value. Because each compartment has two inlets, each inlet carries half of the total flow specified for that compartment. For instance, if the flow rate is set to 10 mL/min per compartment, each inlet provides 5 mL/min. Gravity effects were also considered, with the gravity vector oriented opposite to the inlet fluid direction, and the reference point is set at the cell outlet. A *P*2 + *P*1 discretization scheme was used for the discretization of fluids.

A laminar flow regime was selected based on the Reynolds number (*Re*) calculated at the inlet. At the highest flow rate of 200 mL/min for the two-dimensional (2D) simulations, the *Re* is 2144. This value is below the typical laminar-turbulent transition threshold of *Re* = 2300 for pipe flow, indicating the flow is laminar. For the three-dimensional simulations, the maximum flow rate of 100 mL/min yields a *Re* of 542 in the tube and 294 in the rectangular duct forming the main body of the cell. These values support the use of the Laminar Flow interface in the simulations. Further details on the Reynolds number calculations can be found in the Supporting Information.

### 2.3 Mass Transport

Mass transport simulations were conducted using the Transport of Diluted Species interface in the Chemical Species Transport (CST) module of COMSOL Multiphysics version 6.2. The governing equations employed are Fick’s first law (eq 3) and the mass conservation equation (eq 4), as follows:

$$\vec{\Phi_{i}}=-D_{i}\nabla c_{i}$$ 3

$$\frac{\partial c_{i}}{\partial t}+\nabla \cdot \vec{\Phi_{i}}+\vec{u}\cdot \nabla c_{i}=R_{i}$$ 4

Here, *c*_*i*_ is the concentration of species *i* (mol/m^3^), *D*_*i*_ is the diffusion coefficient of species *i* (m^2^/s), and $\overset{\to }{\Phi_{i}}$ is the diffusive flux of species *i* (mol/(m^2^·s)). The additional terms in eq 4 account for $\vec{u}$ , the fluid velocity (m/s), and *R*_*i*_, the consumption or generation rate of species *i* (mol/(m^3^ · s)). Equation 3 describes mass transport by diffusion, driven by the concentration gradient (chemical potential difference) of species *i*, while eq 4 ensures mass conservation for each species. The diffusion coefficients for glycerol and DHA were obtained from data reported in our previous work,^13^ which provides the diffusion coefficients of GOR products across various glycerol concentrations and temperatures. For the case of 0.5 M glycerol solution at 25 °C, the diffusion coefficients are *D*_GLY_ = 9.31 × 10^–10^ m^2^/s, *D*_DHA_ = 9.67 × 10^–10^ m^2^/s and *D*_H2_ = 2.2 × 10^–9^ m^2^/s.

An important note regarding these equations is that the fluid velocity *u* was used to couple the CFD and CST modules. In other words, the fluid velocity from the laminar flow served as the convective velocity in the mass transport equations. Additionally, each species was assumed to behave ideally, meaning its activity was considered equal to its concentration. Quadratic discretization was used for the concentration. For the 2D simulations, mass transport boundary conditions were set to zero flux at all boundaries except the inlet and outlet. In the 3D simulations, an additional flux originating from the electrode surface was included. For more details regarding the boundary conditions refer to Figure S5.

To investigate product crossover, we utilized the Electrode Surface Coupling physics within the Transport of Diluted Species interface (CST module), assuming a constant current density (*J*) of 10 mA/cm^2^ under which the GOR occurs at the anode and the hydrogen evolution reaction (HER) occurs at the cathode. To relate the concentration of species to the charge, Faraday’s law of electrolysis is used (eq 5).

$$R_{i}=\frac{\nu_{i}J}{nF}$$ 5

where *R*_*i*_ is the consumption or generation rate of species *i* (mol/(m^2^·s)), ν_*i*_ is the stoichiometric coefficient of species *i*, *n* is the number of electrons involved, and *F* is the Faraday constant (96,485 C/mol). The molar flux *R*_*i*_ is set at the upper surface of each compartment to simulate the consumption of glycerol and formation DHA (blue surface) and H_2_ (green surface) at the anode and cathode (Figure S5). The study of crossover coming from the (photo)­electrochemical reactions can only performed using the 3D geometry as any other cross section does not include all the necessary elements to perform this study (e.g., the 2D cross section used for the study on fluid properties does not include the electrode surface in it). All simulations examining the influence of fluid properties on flow behavior were conducted using a 2D geometry, whereas simulations focused on crossover phenomena were performed with a 3D geometries. For the latter, all the results are displayed with a top view toward xy plane (Figure 2d).

### 2.4 Electrochemistry

To quantify the voltage loss arising from ohmic resistance, electrochemical simulations were performed on a 2D *x–z* slice that passes through the bridge (Figure 2a,c). The electrolyte was treated as an aqueous NaNO_3_ solution containing only two ionic species—Na^+^ and NO_3_^–^—so that all charge transport is carried by these ions. Electroneutrality is enforced everywhere:

$$\sum_{i}z_{i}c_{i}=0$$ 6

with *z* _Na+_ = +1 and *z*_NO3–_ = −1. Because the liquid was assumed stagnant and nonreactive, mass conservation reduces to the steady-state Nernst–Planck form:

$$\nabla \cdot J_{i}=0$$ 7

where the molecular flux of species *i* is

$$J_{i}=-D_{i}\nabla c_{i}-z_{i}u_{m,i}Fc_{i}\nabla \varphi_{l}$$ 8

Here *D*_*i*_ is the diffusion coefficient, φ_*l*_ is the electrolyte potential. The ionic mobility *u*_*m*, *i*_ is related to *D*_*i*_ by the Nernst–Einstein relation:

$$u_{m,i}=\frac{D_{i}}{RT}$$ 9

The electrolyte current density *i*_*l*_ is then obtained from

$$i_{l}=F\sum_{i}z_{i}J_{i}$$ 10

### 2.5 Euler–Euler Model

The investigation of the fluid dynamic properties of a mixture of liquid and dispersed gas bubbles was performed using the Euler–Euler model. Here, the continuous phase and the dispersed phase are treated as interpenetrating continua. The volume fractions of the continuous phase (α_*c*_) and the dispersed phase (α_*d*_) satisfy:

$$\alpha_{c}+\alpha_{d}=1$$ 11

The local mass conservation for each phase *k* ∈ {*c*, *d*} is described by the continuity equation:

$$\frac{\partial (\alpha_{k}\rho_{k})}{\partial t}+\nabla \cdot (\alpha_{k}\rho_{k}u_{k})=0$$ 12

where ρ_*k*_ and ***u*** *_k_* denote the density and velocity of phase *k*. Momentum conservation for each phase is governed by

$$\alpha_{k}\rho_{k}\left(\frac{\partial u_{k}}{\partial t}+(u_{k}\cdot \nabla )u_{k}\right)=-\nabla \cdot (pI+\tau_{k})+M_{k}+\alpha_{k}\rho_{k}g$$ 13

where ***I*** is the identity tensor. The viscous stress tensor of phase *k*, τ_*k*_, is written

$$\tau_{k}=\mu_{k}^{m}\left(\nabla u_{k}+(\nabla u_{k}{)}^{T}-\frac{2}{3}(\nabla \cdot u_{k})I\right)$$ 14

where μ_*k*_^*m*^ is the mixture (effective) viscosity. The interfacial momentum exchange term in eq 13, ***M*** *_k_*, is introduced through

$$M_{k}=\frac{m_{dc}}{\alpha_{k}}(u_{d}-u_{k})$$ 15

The momentum-transfer coefficient *m*_*dc*_ is supplied by the Schiller–Naumann drag relation

$$m_{dc}=\frac{3}{4}C_{D}\frac{\rho_{c}\alpha_{d}}{d_{b}}|u_{d}-u_{c}|$$ 16

where *d*_b_ = 100 μm is the bubble diameter and *C*_D_ is the drag coefficient evaluated from the bubble Reynolds number *Re*_b_ < 103 via the Schiller–Naumann correlation:

$$C_{D}=\frac{24}{{Re}_{b}}(1+0.15{{Re}_{b}}^{0.687})$$ 17

## 3 Results and Discussion

### 3.1 Mesh-Independence Study

In finite element method (FEM) simulations, it is crucial to ensure that the results accurately and reliably represent the physical behavior of the system without being influenced by numerical discretization errors. While finer meshes reduce these errors, they also increase computational time exponentially. Therefore, finding a balance between computational cost and accuracy is needed. This balance is achieved through a mesh-independence study, in which the mesh is systematically refined while key solution parameters are monitored. Figure S2 shows the two-dimensional (2D) CFD simulation results for the OHPERA device with various mesh sizes, with pure water as the working fluid. COMSOL Multiphysics offers various automatic mesh size options, ranging from “Coarser” to “Extremely Fine.” Figure S2 highlights the importance of mesh-independence studies: different flow dynamics are observed depending on the mesh size, despite identical conditions being set, in particular the fixed inlet flow rate of 25 mL/min. When the “Coarser” and “Coarse” mesh options are used, the results indicate that the anolyte (in the left compartment) flows into the catholyte, causing a significant undesired mixing of the two electrolytes. However, starting from the “Normal” mesh option, the flow patterns converge to the same behavior: the inlet jet streams merge into a larger jet confined within each compartment, thereby significantly reducing mixing. Among the options tested, the “Fine” mesh was selected as it provided the optimal balance between computation time and accuracy.

### 3.2 Two-Dimensional CFD Simulations

In FEM simulations, it is advantageous to first examine the convergence and physical validity of a simplified model before progressively increasing the complexity and dimensionality of the model. Based on this principle, the anolyte and catholyte flow chambers of the OHPERA device were initially modeled in two dimensions, as shown in Figure 3. The density and dynamic viscosity (hereinafter viscosity) of the fluid are key variables that determine its velocity and pressure profiles (eqs 1 and 2). These properties for an aqueous solution of glycerol are determined by the concentration of the solute and temperature, as shown in Figure S1.^13^ Even if the temperature is assumed to be constant, the concentration of glycerol changes during device operation due to the GOR occurring at the anode and the glycerol supply through the inlet. Consequently, the density and viscosity of the solution also vary over time. Incorporating these changes into the simulation increases the model’s nonlinearity and may negatively impact computational convergence. Therefore, we investigated the conditions under which the glycerol solution could be approximated as pure water. At 25 °C, density changes only slightly as the glycerol concentration increases from 0 M (pure water) to 14 M, ranging from 0.96 to 1.27 g/cm^3^ (Figure S1). The density of a 0.5 M glycerol solution is about 1.007 g/cm^3^ at 25 °C, which is just 1% higher than that of pure water, leading us to initially expect similar fluid behavior. In contrast, the viscosity spans several orders of magnitude as the solute concentration increases and exhibits a pronounced, nonlinear dependence (Figure S1). For example, at 25 °C, the viscosity of pure water is 8.87 × 10^–4^ Pa·s, whereas that of a 0.5 M glycerol solution is 9.97 × 10^–4^ Pa·s—an increase of approximately 12%.

**Figure 3:**
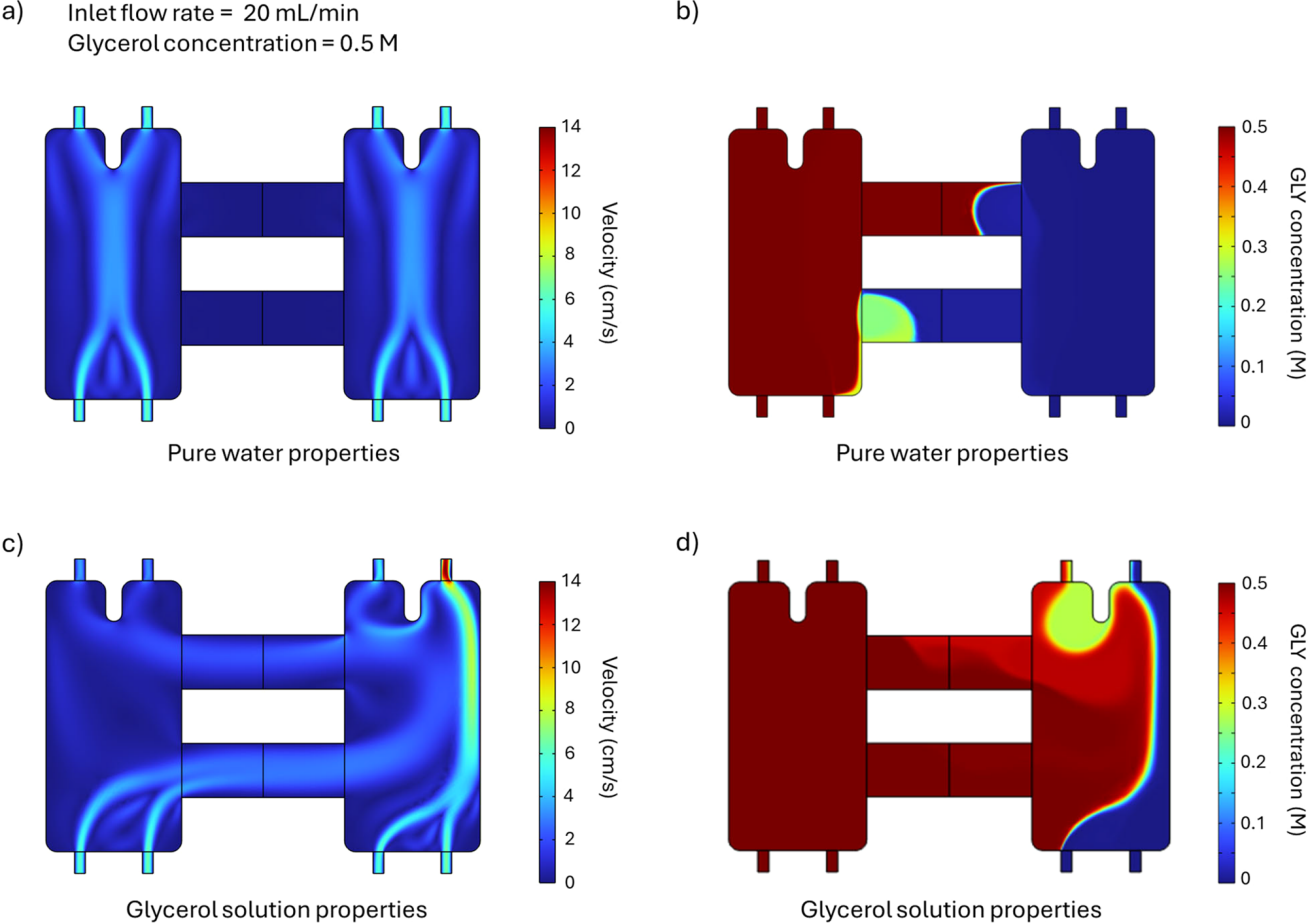
Two-dimensional (2D) fluid velocity and concentration profiles, at a 20 mL/min inlet flow rate, calculated using the density (ρ) and dynamic viscosity (μ) of (a,b) pure water and (c,d) a 0.5 M glycerol solution. The density and dynamic viscosity of a 0.5 M glycerol solution vary with glycerol concentration. The anolyte (0.5 M glycerol) enters the left compartment through its two lower inlets, and the catholyte (water) enters the right compartment through its two lower inlets. The liquids exit through the top outlets in each compartment, which are separated by a flow divider fin.

2D CFD simulations were performed to assess the possibility of approximating the density and dynamic viscosity of the glycerol solution to those of pure water at low glycerol concentrations values (∼0.5 M) and 25 °C. Figure 3a,b and c,d compare the results of the simulations obtained using two different sets of fluid properties: those of pure water (Figure 3a,b) versus those of a 0.5 M glycerol solution, as defined in Figure S1 (Figure 3c,d). The flow rate is set to 20 mL/min in both simulations, glycerol solution is fed to the left compartment, and pure water is fed to the right compartment. When the fluid properties are assumed to be those of pure water (Figure 3a), the inlet jets merge to form a single, wider stream, which then splits near the outlets due to the upper flow divider fin. Additionally, the velocity in the two bridges is nearly zero, suggesting minimal crossover between the anolyte and catholyte. In contrast, when the fluid properties are based on those of 0.5 M glycerol solution (Figure 3c), the anolyte crosses over the catholyte compartment, pushing the catholyte toward the right wall and causing significant mixing. Due to the differences in the results shown in Figure 3a,c, the glycerol solution cannot be approximated as pure water when it is used as the anolyte and pure water is used as the catholyte; instead, its density and viscosity must be defined as functions of glycerol concentration. This phenomenon arises from the coupled transport of mass and momentum. A concentration gradient develops between the anolyte and catholyte, driving glycerol diffusion toward the cathode and water diffusion toward the anode (Figure 3b). As diffusion changes the local composition, it also modifies the fluid’s density and viscosity within the system. When assuming constant properties (i.e., those of pure water), these changes have no impact, and mass transport remains diffusion-dominated. However, when composition-dependent viscosity and density are considered, spatial gradients in fluid properties lead to flow instabilities that enhance mixing, thereby accelerating the reduction of the concentration gradient through convective effects (Figure 3d). As will be discussed in the following section, this enhanced mixing is primarily driven by density gradients between water and the 0.5 M glycerol solution, rather than by variations in viscosity.

To determine whether the density and/or the viscosity of the glycerol solution causes the observed different flow behavior compared to pure water, we conducted CFD simulations under three conditions at various flow rates. First, the properties of pure water were employed for the fluid on both the anolyte and catholyte (Figure 4a). At a low flow rate of 20 mL/min, the velocity in the two bridges connecting the anolyte and catholyte compartments is below 0.1 cm/s, indicating minimal crossover between the two compartments. As the flow rate increases, the velocity in the two bridges also rises, and at flow rates above 100 mL/min, a distinct stream appears in these bridges.

**Figure 4:**
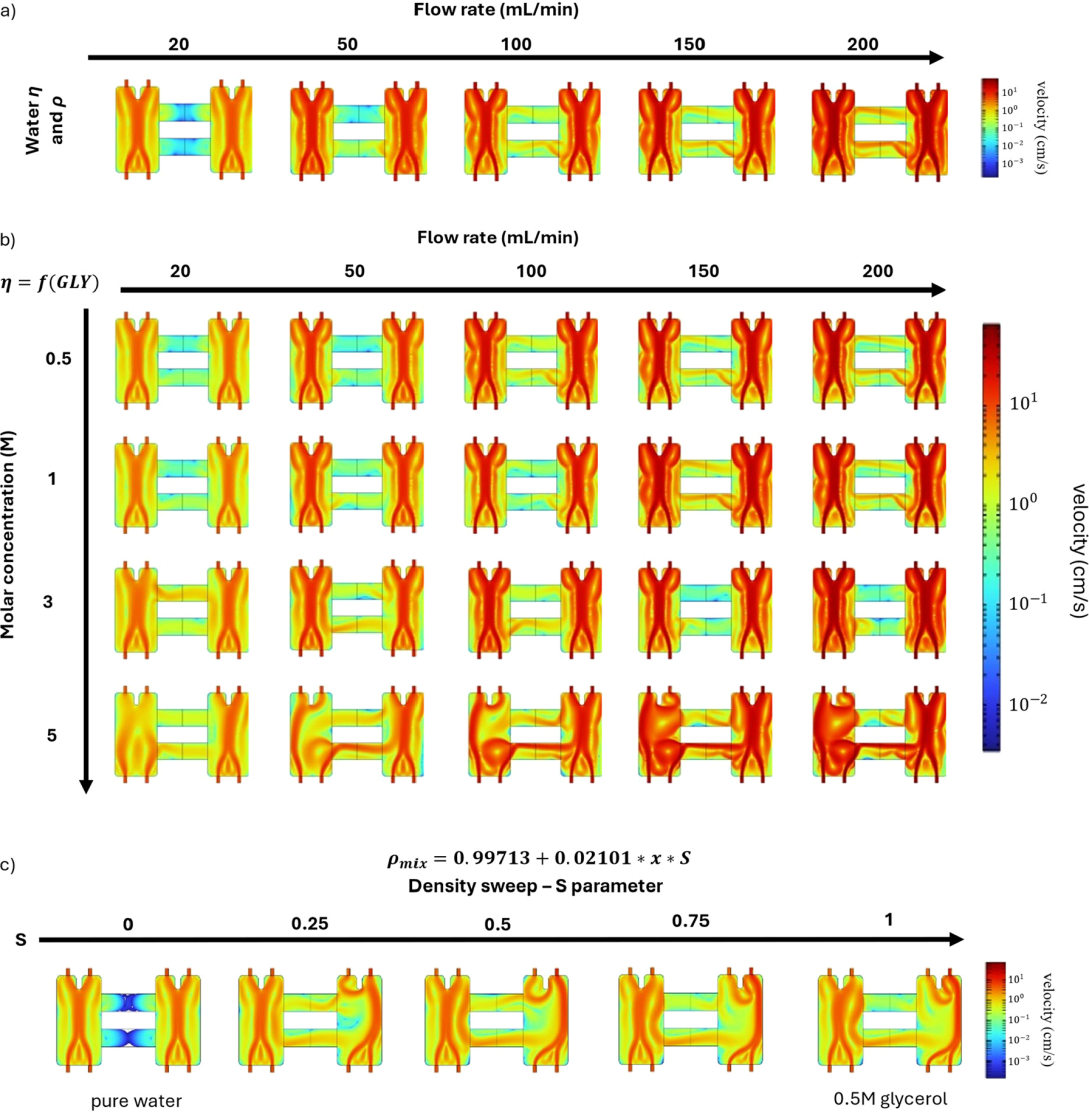
(a) Two-dimensional (2D) fluid velocity profile at different flow rates (20–200 mL/min), calculated using the density and dynamic viscosity of pure water. (b) 2D fluid velocity profile at various glycerol concentrations (0.5–5 M) and flow rates (20–200 mL/min), calculated using the density of pure water and the viscosity of the glycerol solution at the corresponding concentration. (c) 2D fluid velocity profile of a 0.5 M glycerol solution at a flow rate of 20 mL/min, calculated using the density of the glycerol solution and the viscosity of pure water. A logarithmic scale is used for the color map to highlight small changes in flow behavior. Note that the anolyte is in the left compartment, while the catholyte is in the right compartment. The liquid enters the compartments through the two lower inlets and exits through the top outlets, which are separated by the flow divider fin.

Second, the fluid was assumed to have the density of pure water, but the viscosity depends on glycerol concentration. Figure 4b shows the CFD simulation results for various solute concentrations and flow rates. The viscosity of the 0.5 and 1 M glycerol solutions are 9.97 × 10^–4^ and 1.12 × 10^–3^ Pa·s, respectively, which represent approximately 12 and 26% differences compared to pure water. Nonetheless, for glycerol concentrations up to 1 M, the fluid flow remains comparable to that of pure water. Noticeable differences emerge at a solute concentration of 3 M. For example, at a flow rate of 20 mL/min, a distinct stream forms in the two bridges (which was not observed with pure water). Conversely, at 200 mL/min, this stream becomes less pronounced—whereas in the case of pure water, it stays clearly visible. Even so, within the anolyte and catholyte compartments, the flow appears similar to that of pure water. Given that the viscosity of the 3 M glycerol solution is 1.85 × 10^–3^ Pa·s—nearly twice that of pure water—one may infer that viscosity alone has only a modest effect on the overall flow behavior.

Third, the fluid was assumed to have the density of the glycerol solution but the viscosity of pure water (Figure 4c). To address convergence issues, a parametric study was conducted at an inlet flow rate of 20 mL/min. This study involved gradually increasing the solution’s density from that of pure water (0.99713 g/cm^3^) to that of a 0.5 M glycerol solution (1.00755 g/cm^3^) using a parameter called *S* (eq 18):

$$\rho (c_{\mathrm{GLY}},S)=0.99713+0.02101\cdot c_{\mathrm{GLY}}\cdot S$$ 18

When *S* = 0, the density of the glycerol solution is set equal to that of pure water, whereas for *S* = 1, the density of the medium corresponds to that of a 0.5 M glycerol solution. Note that the properties of the catholyte’s medium remain those of pure water in all cases. Due to convergence issues, a “Fine” or “Extremely Fine” mesh was used in this study. As shown in Figure 4c, increasing the value of *S* raises the velocity of the streams moving toward the catholyte, eventually altering the flow pattern, such that the main liquid stream is pushed toward the chamber wall. Since both the “Fine” and “Extremely Fine” meshes produce the same flow pattern, and numerical error decreases with finer mesh resolution, we conclude that the crossover is not driven by numerical artifacts. Instead, the results suggest that, despite the relatively small difference in density between pure water and 0.5 M glycerol solution (only 1%), this slight difference is the primary cause of the observed crossover.

We investigated whether increasing the flow rate could reduce the anolyte’s liquid crossover into the catholyte. Unfortunately, even at a high volumetric flow rate approaching the device’s operational limit, glycerol crossover into the catholyte still persists (Figure S6). Therefore, when glycerol is present only in the anolyte, preventing crossover without a membrane appears challenging. An alternative solution for a membraneless device is to use the same glycerol-containing solution for both the anolyte and the catholyte. This approach primarily prevents mixing caused by the difference in density between the two fluids. It also relies on our previous observation that glycerol does not react with the hydrogen generated at the cathode.^12^ Therefore, for the 3D simulations discussed below, we assume that both the anolyte and catholyte are glycerol solutions with the same concentration. Additionally, it is no longer necessary to include any concentration-dependent changes in density and viscosity. From this point onward, the fluid properties are taken to be those of a 0.5 M glycerol solution—1.007 g/mL and 9.97 × 10^–4^ Pa·s, respectively, as determined by Figure S1.

One might question whether the density and viscosity should be recalculated as glycerol is consumed at the anode via the GOR. However, glycerol is not simply removed; it is converted into another compound, so the total mass remains the same and, thus, the overall density does not change. For example, when one glycerol molecule (molecular weight 92 g/mol) is oxidized to DHA (approximately 90 g/mol) while generating two protons (1 g/mol each), the total mass—and therefore the density—remains constant. Although protons diffuse roughly ten times faster than glycerol, causing a minor local difference in density, this effect should be minimal under flowing conditions. Likewise, viscosity may vary with glycerol concentration, but for the sake of simplicity in our model, we treat it as a constant.

### 3.3 Three-Dimensional Simulations Coupling Fluid Dynamics and Mass Transport

We have demonstrated that using a glycerol solution as both the anolyte and the catholyte can minimize undesirable mixing between the two compartments without a membrane. As previously mentioned, this approach relies on the assumption that the presence of glycerol does not affect the hydrogen evolution reaction at the cathode and does not react with the hydrogen produced there. However, in the absence of a membrane, there remains a possibility of crossover between the GOR products and hydrogen. Since effective product separation is crucial for enhancing the economic value of our PEC device, minimizing such crossover is essential. To investigate this, we incorporated the generation of GOR products, protons, and hydrogen into our model. This was carried out using the Electrode Surface Coupling physics within the Transport of Diluted Species interface, described in more detail in the Methods section. We assumed a current density of 10 mA/cm^2^ at the anode and −10 mA/cm^2^ at the cathode, corresponding to the target current density of the OHPERA project. At the anode, glycerol is oxidized to DHA with 100% Faradaic efficiency, producing two protons (H^+^) according to the reaction below (eq 19):

$$C_{3}H_{8}O_{3}\to C_{3}H_{6}O_{3}+2H^{+}+2e^{-}$$ 19

At the cathode, likewise with 100% Faradaic efficiency, two protons are reduced to form one hydrogen molecule (eq 20):

$$2H^{+}+2e^{-}=H_{2}$$ 20

3D multiphysics simulations, which couple fluid dynamics and mass transport, were conducted at flow rates ranging from 10 to 100 mL/min, with a 0.5 M glycerol solution supplied to both the anolyte and catholyte. Figure S6 shows the fluid pattern at a flow rate of 30 mL/min. The highest velocities occur at the inlets, followed by the formation of jets as the fluid enters the main body of each compartment. As these jets progress through the cell, they lose momentum and do not completely mix with the surrounding fluid, thus maintaining a continuous flow path from the inlet to the outlet. Between these jets and along the cell walls, areas of low fluid velocity appear, with the smallest values occurring near where the fluid enters and exits. In the connecting bridges, the velocity is almost zero. This is desirable for minimizing crossover, as no streams flow from one compartment to the other, thereby reducing mixing.

Figure 5a–c shows the fluid velocity profile (on the *xy*-plane, *z* = 0), DHA concentration (on the device surface), and H_2_ concentration (on the device surface), respectively, at flow rates of 10, 40, 70, and 100 mL/min. As the flow rate increases, the velocity rises and the jet streams become more pronounced, enhancing mass transport and decreasing the diffusion boundary layer. At lower velocities, H_2_ accumulates near the electrode surface, whereas at higher velocities, product accumulation is significantly reduced, resulting in a more uniform concentration near the electrode surface. Consequently, the concentration of products remaining in the cell (Figure 5b,c) decreases markedly with increasing flow rate.

**Figure 5:**
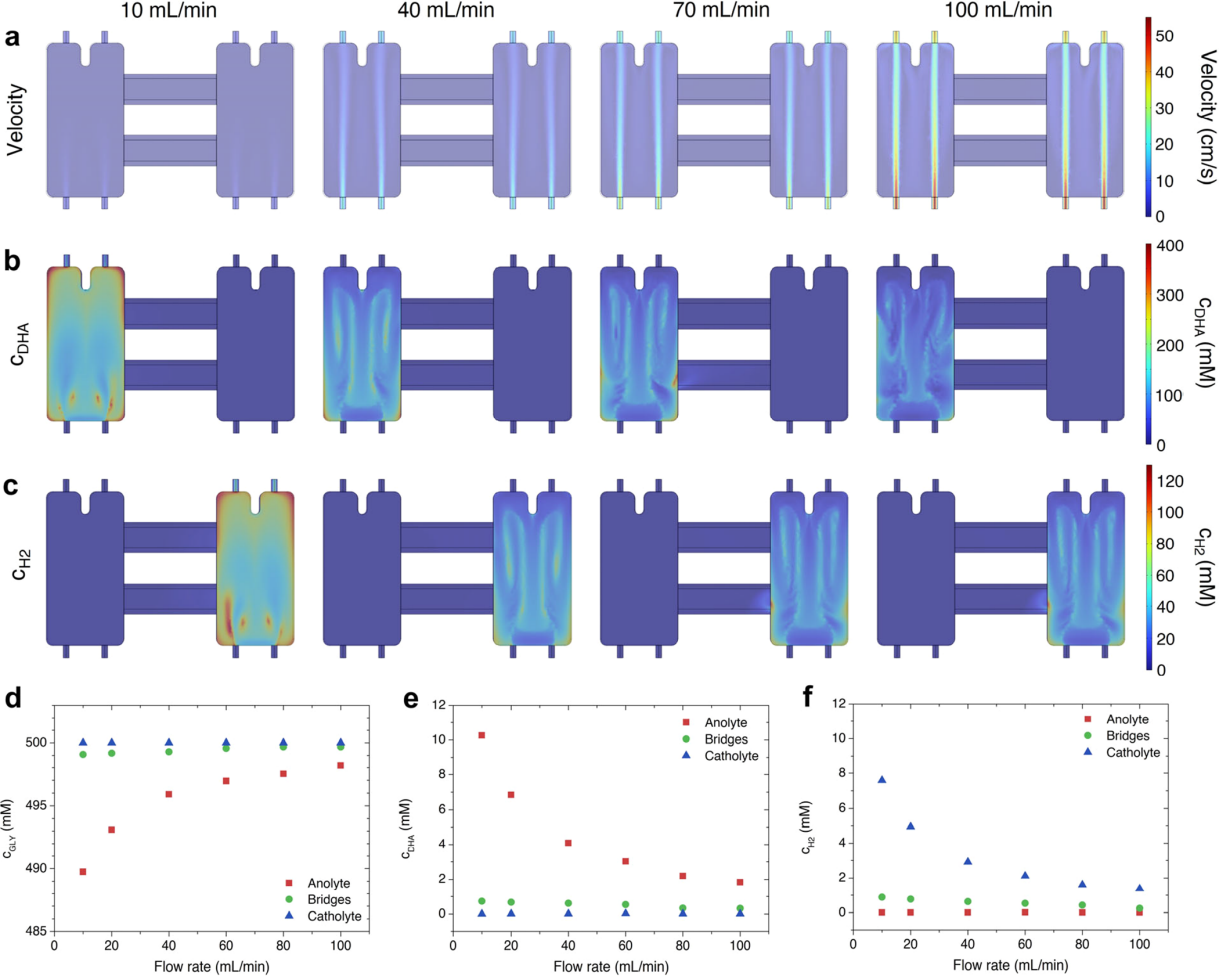
(a) Fluid velocity profile on the *xy*-plane, (b) DHA concentration (*c*_DHA_), and (c) H_2_ concentration (*c*_H2_) on the device surface at flow rates of 10 mL/min (1st column), 40 mL/min (2nd column), 70 mL/min (3rd column), and 100 mL/min (4th column). Volume-averaged (d) glycerol concentration (*c*_GLY_), (e) *c*_DHA_, and (f) cH_2_ in the anolyte (red squares), bridges (green circles), and catholyte (blue triangles).

Figure 5d,e displays the volume-averaged concentrations of H_2_ and DHA in the anolyte (black squares), the bridges (red circles), and the catholyte (blue triangles). It is evident that the concentration of each compartment’s primary product (H_2_ in the catholyte and DHA in the anolyte) declines as the flow rate increases. Additionally, higher flow rates lead to a smaller deviation of the volume-averaged pH from its initial value of zero (Figure 5f). This finding suggests the device should be operated at sufficiently high flow rates, likely at or above 60 mL/min, to avoid significant pH variations that can damage the electrode, affect reaction kinetics, or alter the reaction selectivity.

Noteworthy, although product concentrations in the bridges also decrease with increasing flow rate, they remain relatively significant. For example, at a flow rate of 20 mL/min, the volume-averaged concentration of DHA in the anolyte and in the bridges is 6.85 and 0.665 mM, respectively, about 10%, which is not negligible. Nevertheless, the crossover between the anolyte and catholyte compartments (represented by DHA concentration in the catholyte and H_2_ concentration in the anolyte) remains very low, even at lower flow rates. Hence, the bridges effectively minimize crossover without a membrane, even at lower flow rates.

By using a common glycerol solution for both the anolyte and catholyte while introducing bridges, we have demonstrated that product crossover can be effectively minimized without a membrane. A potential drawback of this bridge-equipped PEC cell, however, is the large *iR* drop. In our earlier work,^12^ the conductivities of 0.5 M electrolyte solutions were measured to be on the order of 100 mS/cm. Assuming spatially uniform conductivity, a current density of 10 mA/cm^2^, and the current bridge dimensions, the bridge alone contributes roughly 5 V of ohmic loss (see Supplementary Note 3 for details). Eliminating the bridges altogether is impractical from a crossover standpoint. As Figure S8 shows, removing the bridges yields ∼2.6% c_H2_-to-c_DHA_ in the anolyte at a flow rate of 50 mL/min.

We therefore retain the bridge concept and explore strategies to reduce its associated voltage penalty through electrochemical simulations. For simplicity, a 2D model representing the *x*-*z* cross-section of the cell was employed. Overpotentials originating from concentration and partial-pressure changes during operation were neglected. The electrolyte conductivity was estimated from the mobilities of Na^+^ and NO_3_^–^, assuming that ion diffusion and migration are the dominant charge-transport mechanisms and neglecting activity-coefficient effects. The detailed methodology is given in the Methods section.

Figure 6a depicts the spatial distribution of the electrolyte potential in the *x–z* plane for four electrolyte concentrations—0.5, 1.0, 1.5, and 2.0 M—referenced to the cathode, which was held at 0 V in the simulation. As the electrolyte concentration increases, the potential gradient across the bridge is visibly flattened. Figure 6b plots the electrolyte potential along a line that traverses the bridge at *y* = 3 mm (indicated by colored dashed lines in Figure 6a). The largest single improvement occurs when the concentration is raised from 0.5 M (3.81 V) to 1.0 M (1.91 V), giving a drop of 1.90 V. A further increase from 1.5 M (1.27 V) to 2.0 M (1.01 V) yields only 0.26 V, indicating diminishing returns at high ionic strength.

**Figure 6:**
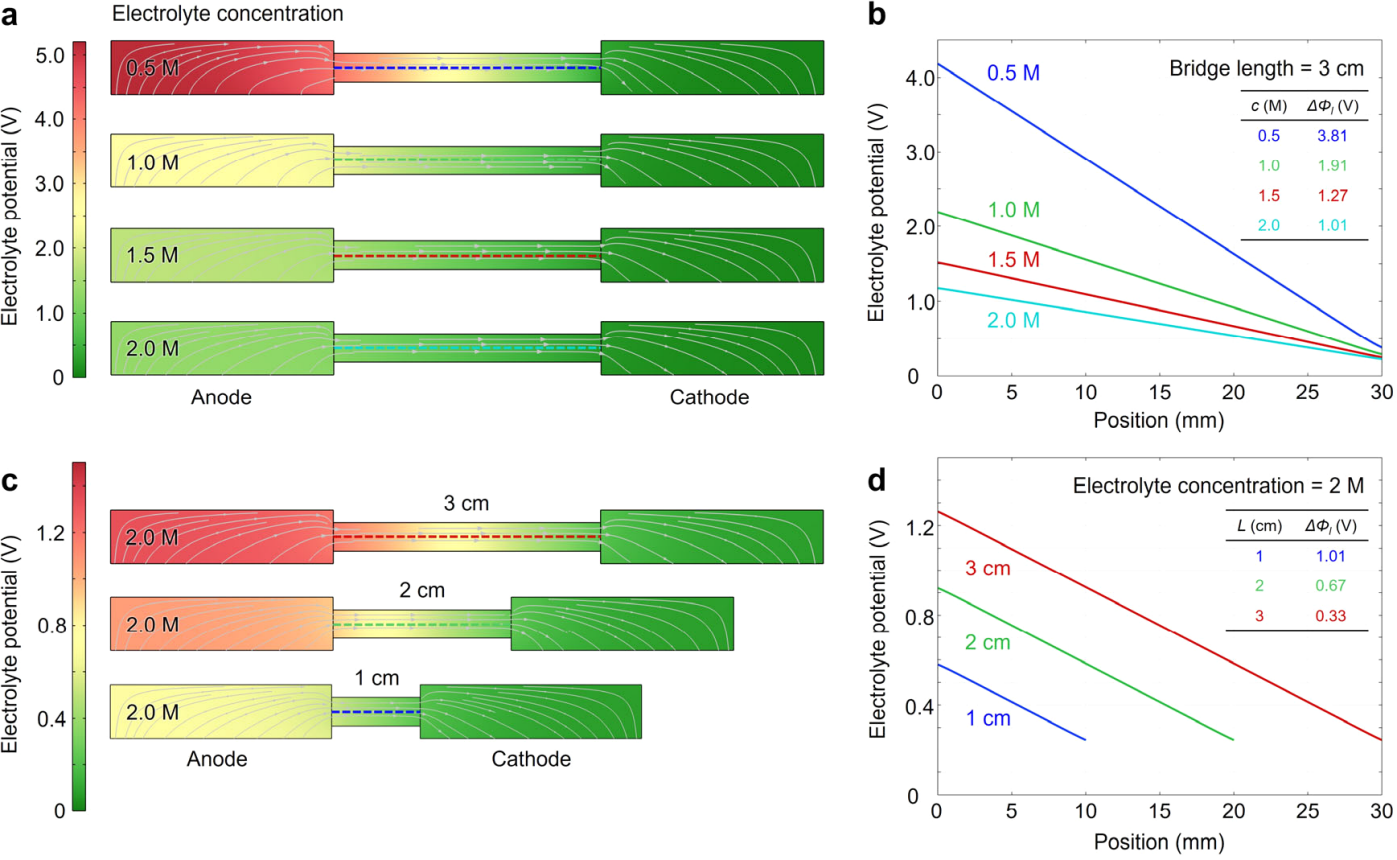
Simulated electrolyte potential distribution and *iR* drop as a function of electrolyte concentration and bridge length (potential referenced to the cathode at 0 V). (a) Color maps of the potential in an *x*–*z* slice for a fixed bridge length of 3 cm at four electrolyte concentrations (0.5, 1.0, 1.5, and 2.0 M). White streamlines trace current paths; the dashed line marks the cross-section (*y* = 3 mm) used in (b). (b) Potential profiles along the dashed line in (a). Increasing the electrolyte concentration (*c*) flattens the gradient and lowers the bridge-related *iR* drop (Δφ_*l*_), as shown in the inset table. (c) Potential maps at 2.0 M for bridge lengths of 3, 2, and 1 cm (cross-sectional area held constant). (d) Corresponding potential profiles. Shortening bridge length (*L*) reduces Δφ_*l*_ from 1.01 V (3 cm) to 0.33 V (1 cm), as shown in the inset table.

These simulations consider only the ionic contribution to conductivity; they do not include changes in viscosity, activity coefficients, or electrode kinetics. Although higher electrolyte concentrations increase bulk conductivity and thus lower the bridge-related *iR* drop, they may also raise the solution viscosity and enhance ion–glycerol interactions^12^ Such effects may reduce the chance of glycerol adsorption on the photoanode surface, potentially offsetting the ohmic gains. Consequently, the optimum electrolyte concentration should be established by coupling the present ohmic analysis with experimental validation of glycerol oxidation performance.

The geometry of the bridge is another major factor that governs the *iR* drop. At a fixed ionic conductivity, the ohmic resistance of the electrolyte within the bridge is directly proportional to the bridge length and inversely proportional to the bridge cross-sectional area, assuming a uniform current density. To isolate the length effect, we held the cross-sectional area constant and kept the electrolyte concentration at 2 M, then shortened the bridge from 3 to 2 cm and finally to 1 cm. The resulting electrolyte-potential maps are shown in Figure 6c. Figure 6d also plots the potential profile along the dashed line in Figure 6c (*y* = 3 mm). As the length decreases from 3 to 2 and 1 cm, the bridge-induced *iR* drop falls from 1.01 to 0.67 and 0.33 V, respectively—approximately two-thirds and one-third of the original value, in line with Ohm’s law. As a result, by combining the higher electrolyte conductivity achieved at 2.0 M with the shorter 1 cm bridge, the total *iR* drop can be reduced from 3.81 V (0.5 M, 3 cm) to 0.33 V (for context, a typical Si-based single junction solar cell can deliver a potential between 0.5 and 0.7 V near the maximum powerpoint of the device).

A potential drawback of the side-by-side PEC cell is the highly anisotropic current distribution that develops in the bridge. Figure 7a presents color maps of the electrolyte current density at an electrolyte concentration of 2 M for the three bridge lengths investigated. Because the anode and cathode face the same direction, the current streamlines must bend sharply to pass through the bridge, which acts as a bottleneck. Although the average cell current density is 10 mA/cm^2^, the local current density inside the bridge rises to about 70 mA/cm^2^. Edge effects are particularly severe at the bridge entrance. Figure 7b plots the current density along the red dotted line at the anolyte-side entrance. Near both bridge edges the current density is several times higher than the midchannel value (∼70 mA/cm^2^); at the lower rim of the bridge (*y* ∼ 1.4 mm)—the shortest path from anode to cathode—it reaches ∼300 mA/cm^2^. This behavior is essentially independent of bridge length, as indicated by the overlapping profiles in Figure 7b.

**Figure 7:**
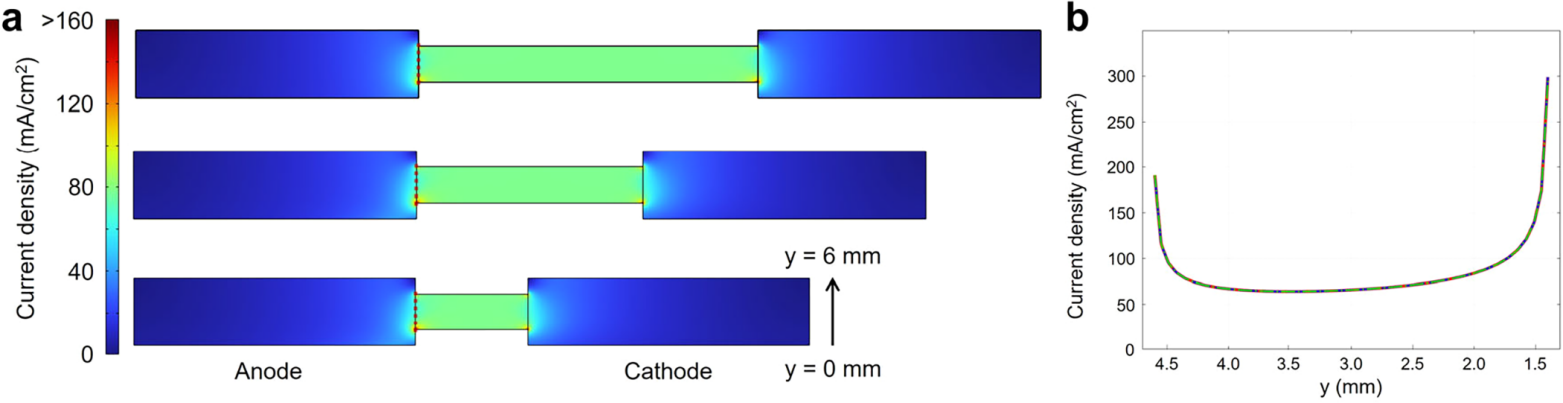
Current distribution in a side-by-side PEC cell (electrolyte concentration = 2 M). (a) Color maps of the magnitude of the electrolyte current density for bridge lengths of 3 cm (top), 2 cm (middle), and 1 cm (bottom). (b) Current-density profiles along the dashed line at the anolyte-side bridge entrance.

Crossover was evaluated in the cell equipped with the short-and-wide bridge (1 cm long, 1.26 cm^2^ cross-section), as shown in Figure 8. At a flow rate of 50 mL/min, the volume-averaged concentration of DHA in the anolyte is 2.92 mM, while that of H_2_ is 0.0281 mM, limiting the H_2_-to-DHA concentration ratio to below 1% (0.96%). In the catholyte, the volume-averaged concentrations of DHA and H_2_ are 0.0231 and 5.07 mM, respectively, corresponding to a crossover ratio of 0.46%. By adopting this new bridge design, our PEC device effectively prevents product crossover without requiring a membrane, while also reducing the voltage loss caused by the bridges.

**Figure 8:**
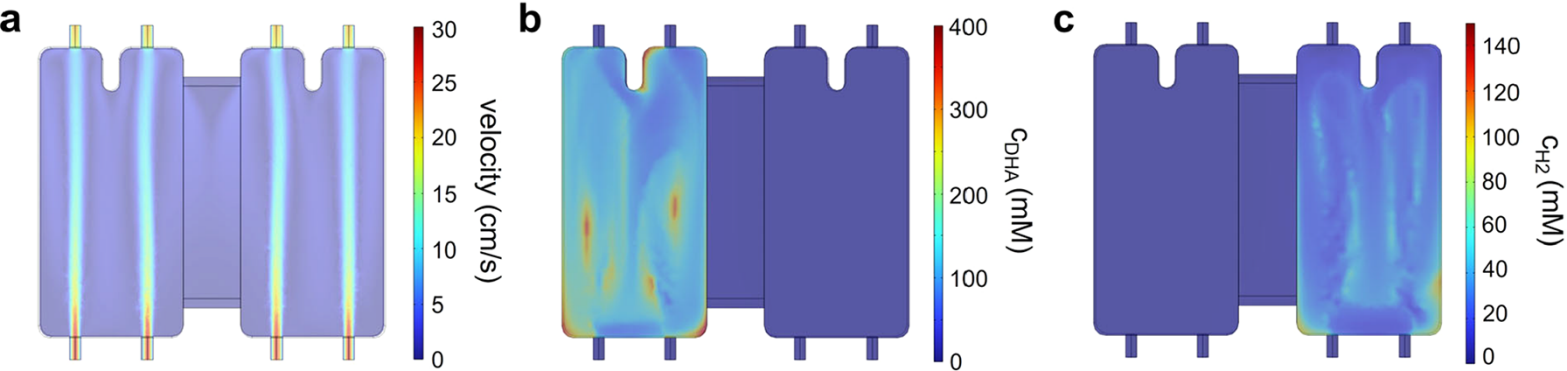
Three-dimensional (3D) simulation results for the device with a single, expanded-area bridge. (a) Fluid velocity, (b) DHA concentration (*c*_DHA_), and (c) hydrogen concentration (*c*_H2_) calculated at a flow rate of 50 mL/min.

### 3.4 Effect of Hydrogen Bubble Evolution on Flow

Figures 5 and 8 analyzed product crossover under the implicit assumption that all hydrogen generated at the cathode remains dissolved in the electrolyte. However, the solubility of hydrogen in water at 25 °C and 1 atm is limited by Henry’s law to about 0.78 mM. Accounting for Henry’s law and the cell operating conditions (current density, flow rate, etc.), we calculated that at most 2.5% of the hydrogen can remain dissolved (see Supplementary Note 4 for details). Hence the remaining 97.5% of the generated hydrogen inevitably leaves as gas bubbles.

We therefore introduced an Euler–Euler two-phase model to assess how these hydrogen bubbles influence the liquid flow and crossover. Because a full 3D simulation proved intractable, we restricted the analysis to a 2D *y*–*z* slice of the catholyte compartment that includes both the inlet and outlet. Figure 9a displays the resulting bubble volume-fraction map: bubbles nucleate on the cathode, rise along its surface, and then follow the adjacent wall toward the outlet. Figure 9b compares velocity-magnitude fields obtained without (left) and with (right) the Euler–Euler model; in the right-hand map, the color scale represents the velocity magnitude of the continuous phase. When bubbles are included, the electrolyte jet bends appreciably, indicating that the dispersed phase exerts a significant drag on the continuous phase. Figure 9c,d shows *y*-velocity profiles at three horizontal cuts (top, mid, bottom; red, green, and blue lines in Figure 9b) without and with the Euler–Euler model, respectively. Without bubbles, a near-parabolic profile is retained throughout the cell; with bubbles, the continuous-phase velocity near the cathode is markedly higher, while backflow (negative velocity) develops in regions farther from the cathode, especially below the midplane.

**Figure 9:**
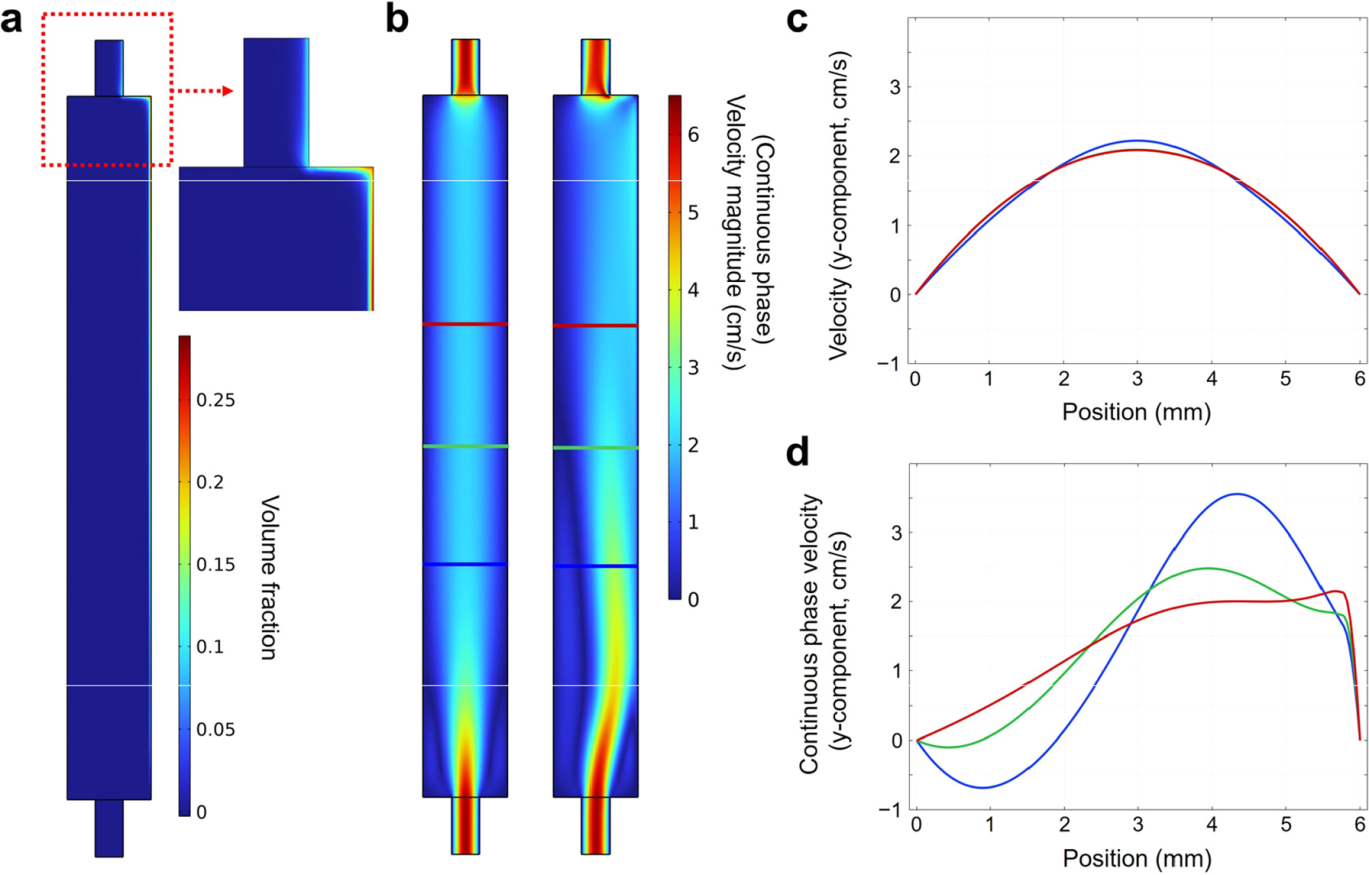
Euler–Euler simulation of hydrogen-bubble evolution in the catholyte compartment (2D *y*–*z* slice). (a) H_2_ bubble volume fraction. (b) Velocity-magnitude fields of the fluid. Left: single-phase (Euler–Euler model disabled). Right: continuous-phase velocity with the Euler–Euler model enabled. (c) *y*-component velocity profiles across the channel at three heights (red, green, and blue lines in (b)) for the single-phase model and (d) corresponding profiles for the continuous phase.

The simulation thus confirms that bubble evolution produces appreciable flow distortion inside the catholyte compartment. Whether these perturbations extend across the bridge into the anolyte—and thereby alter crossover—cannot be resolved with the present 2D model. Attempts to simulate the full 3D geometry with Euler–Euler physics were unsuccessful because of convergence issues, probably caused by the model’s strong nonlinear coupling. Consequently, the ultimate impact of gas evolution on crossover must be clarified experimentally and remains an important topic for future work.

## 4 Conclusions and Outlook

In this study, we systematically investigated the transport properties of a 0.5 M glycerol solution and its implications for the operation of a membraneless PEC device. Our findings highlight critical factors influencing flow behavior, crossover, and device performance, providing valuable insights for scaling up PEC systems for biomass-derived glycerol oxidation and H_2_ production.

- The transport properties of a 0.5 M glycerol solution differ significantly from those of pure water, despite their seemingly similar density and viscosity values. The density variation, albeit small (∼1% increase compared to pure water at 25 °C), significantly alters the flow pattern and induces crossover effects.
- The viscosity of the glycerol solution also influences flow characteristics, increasing by approximately 12% at 0.5 M compared to pure water (9.97 × 10^–4^ Pa·s vs 8.87 × 10^–4^ Pa·s at 25 °C). However, our simulations indicate that density-driven effects dominate over viscosity-driven changes in determining flow separation and electrolyte mixing.
- Operating the PEC device with 0.5 M glycerol solution in the anolyte and pure water in the catholyte leads to severe mixing and crossover due to density-driven instabilities. Conversely, using the same glycerol-containing solution in both compartments eliminates undesired mixing and enables effective membraneless operation.
- In our 3D simulations, we analyzed crossover effects for a current density of 10 mA/cm^2^ and found that, at high flow rates (≥60 mL/min), product crossover remained negligible. Even at 20 mL/min, the concentration of DHA in the catholyte was just ∼0.665 mM (about 10% of its concentration in the anolyte), confirming the effectiveness of our bridge design in limiting product mixing.
- Raising the electrolyte concentration and shortening the bridge substantially reduced the bridge-related *iR* drop, minimizing the principal voltage penalty of the side-by-side device while preserving membrane-free operation.
- Nevertheless, the device geometry inherently yields a highly anisotropic current distribution, creating persistent hot-spots even with the shorter bridge and indicating that additional measures may be needed to limit local Joule heating. This is of primarily importance especially in the future scaling up of the device, where the spatial distribution of the current density plays a crucial role on the overall energy efficiency of the device.^31^
- Two-phase (Euler–Euler) simulations show that hydrogen bubbles markedly disturb catholyte flow. However, full 3D modeling is computationally prohibitive, so the ultimate impact of bubble dynamics on crossover must be determined experimentally.

Scaling up PEC devices for biomass reforming, particularly glycerol oxidation, presents a promising pathway for sustainable hydrogen and, in particular, value-added chemical production.^1^ Our findings support the feasibility of membraneless PEC configurations, which reduce material costs and can improve operational stability compared to membrane-based systems. With an optimized flow configuration and bridge geometry, our system minimizes product crossover while maintaining a target current density of 10 mA/cm^2^. Higher flow rates enhance mass transport, reduce boundary layer effects, and improve reactant availability at the electrode surface. A modular approach, where multiple PEC units operate in parallel, can increase throughput while maintaining efficiency. Future studies should explore device designs that maximize solar-to-chemical conversion efficiency (i.e., Solar-to-X efficiency), such as tandem PEC cells with improved light absorption,^1,32^ and further reduce ohmic voltage losses in the electrolyte. Real-world operation under solar illumination will be crucial for proving the effectiveness of our models and to assess the technological readiness of solar-driven biomass reforming.

## Supplementary Material



## Notes

Technical specifications of the device, calculation of the Reynolds number and *iR* drop for the liquid lines and the bridge connecting the two compartments of the device, estimation of the dissolved hydrogen fraction in the catholyte for a current density of 10 mA/cm^2^, additional numerical calculations regarding the mesh independence, description of the boundary conditions implemented in the simulations, additional fluid dynamics simulations as a function of volumetric flow rate, and three-dimensional simulations for the device without a connecting bridge (PDF)
