# Supplementary material for: Multiphysics Modeling of Photoelectrochemical Devices for Simultaneous Solar-Driven Biomass Reforming and Hydrogen Production
Source: Energy Fuels. 2025 Jun 18;39(26):12716–30. doi: 10.1021/acs.energyfuels.5c01590 (PMC12235749; doi:10.1021/acs.energyfuels.5c01590)
Supplement: Supplementary file 1 [file ef5c01590_si_001.pdf]

Supporting Information

Multiphysics Modeling of Photoelectrochemical Devices for Simultaneous Solar-Driven Biomass Reforming and Hydrogen Production

Andrés F. Pérez Torres[†1,2], Heejung Kong[†1,2], Senapati Sri Krishnamurti[1], Feng Liang[1], Sixto Giménez[3], Roel van de Krol[1,2], Marco Favaro[*1]

[1] Institute for Solar Fuels, Helmholtz-Zentrum Berlin für Materialien und Energie GmbH, Hahn-Meitner-Platz 1, 14109 Berlin, Germany.

[2] Institut für Chemie, Technische Universität Berlin, Straße des 17. Juni 124, 10623 Berlin, Germany.

[3] Institute of Advanced Materials, Universitat Jaume I, Avinguda de Vicent Sos Baynat, s/n, 12006 Castelló de la Plana, Spain.

[†] A.F.P.T. and H.K. contributed equally to this paper.

* Author to whom correspondence should be addressed: M. Favaro (marco.favaro@helmholtz-berlin.de)

KEYWORDS. Multiphysics modeling, finite element method, photoelectrochemistry, photoelectrochemical devices, glycerol oxidation, hydrogen production, fluid dynamics, Navier-Stokes equations.

Supplementary Note 1. Technical specifications of the device

The device features the following technical specifications:

- Double-cassette peristaltic pump will be used for precise flow rate synchronization of the two compartments.
- The PEC cell will be 3D printed using process compatible, low-cost, halogen-free, and recyclable materials such as acrylonitrile styrene acrylate (ASA, cost per one unit € 18.75) and polycarbonate (PC, cost per one unit € 34.45). Both materials are compatible with the common fused deposition modelling (FDM) and multi-jet modelling (MJM) 3D printing technique.
- The gaskets used for the PEC cell sealing are made in fluoroelastomer rubber (Viton).
- Sensors will be installed in the inlet tubes just before the PEC cell, for in-line measurements of the volumetric flow rate, conductivity of the flowing electrolyte, and temperature, on a timescale spanning from 1 second to hundreds of hours.
- Fluid capacitors will be installed in the inlet tubes for each compartment to damp the typical peristaltic pulsation of the flowing liquid[1,2].
- All ports are UNF(F) ¼-28 Flat bottom (Imperial gauge).
- Liquid lines: 1/8" OD (~ 3.2 mm) / .085" ID (~ 2.2 mm).
- Laminar flow for pure water in the liquid lines up to about 200 mL/min (considering pure water and a Reynolds number, Re, ≤ 2500).
- The PEC cell features two additional ports on both anolyte/catholyte for local sensing (pH, temperature, Clark electrode, etc.)[1].

- Each compartment of the PEC cell hosts a support that can be used for mounting an M8-threaded supporting shaft. Additionally, each compartment is equipped with a flow-separating fin on the cell outlet to avoid turbulence.

Supplementary Note 2. Calculation of the Reynolds number

To determine the appropiate interface for the computational fluid dynamics simulations (CFD), the Reynolds number (Re) through the inlet tubes was calculated as follows:

$$Re = \frac{\rho\, u\, L}{\mu}$$

Where ρ is the density in of the fluid (kg/m^3), u the velocity (m/s), L the characteristic length in (m) and μ the dynamic viscosity of the fluid ($Pa \cdot s$). The density and dynamic viscosity are those of 0.5 M glycerol solution with a value of 1.007 g/cm^3 and 9.97×10^{-4} Pa·s, respectively. The characteristic length for the inlet tubes is their diameter (0.002 m), while for each compartment that makes up the cell, is the hydraulic diameter:

$$D_h = \frac{2ab}{a + b}$$

Here a and b correspond to the length and heigh of the rectangle formed by the cross section of the rectangular duct. **Table S1** contains the initial estimated *Re* using the average velocity (*u)* within the tubes, calculated by using their cross-section area (*A*) and the flow rate (*Q*):

$$u = \frac{Q}{A}$$

Supplementary Note 3. Calculation of the *iR* drop

Given an electrode area of 10 cm^2 and a current density of 10 mA/cm^2 (0.01 A/cm^2), the total current is 0.1 A. The conductance G (S or 1/Ω) of the electrolyte, considering two bridges in parallel, each with length L (cm), total cross-sectional area A (cm^2), and electrolyte conductivity κ (S/cm), is given by:

$$G = \frac{\kappa L}{A}$$

and resistance R (Ω) is the reciprocal of G:

$$R = \frac{1}{G}$$

Given the electrolyte conductivity κ of the 0.5 M electrolyte solution of 100 mS/cm, the conductance G of the bridges is approximately 0.02 S, and the resistance R is around 50 Ω. Consequently, the *iR* drop through the bridges is:

$$0.1\, A \times 50\, \Omega = 5\, V$$

Thus, under these conditions, the calculated *iR* drop across the bridges is approximately 5 V.

Supplementary Note 4. Estimation of dissolved hydrogen fraction

To determine the proportion of hydrogen that can remain dissolved, we compare the total amount of hydrogen generated electrochemically with the solubility-limited amount of hydrogen that can be retained in the liquid phase. The rate of hydrogen generation (r_{gen}) is given by Faraday's law:

$$r_{gen} = \frac{i \cdot A}{2F} = 5.18 \times 10^{-6}\ mol/s$$

where i = 10 mA/cm^2 is current density, A = 10 cm^2 is the electrode area, F = 96,485 C/mol is the Faraday's constant, and the factor of 2 accounts for the two electrons required to produce one hydrogen molecule. According to Henry's law, the solubility of hydrogen in water at 25 °C and 1 atm is $c_{H2,\ max}$ = 0.78 mM. Given an electrolyte flow rate of Q = 10 mL/min, the maximum rate at which hydrogen can remain dissolved, r_{dis}, is:

$$r_{dis} = c_{H2,\ max} \cdot Q = 1.30 \times 10^{-7} mol/s$$

Then, fraction of hydrogen that can remain dissolved is

$$\frac{r_{dis}}{r_{gen}} = \frac{1.30 \times 10^{-7} mol/s}{5.18 \times 10^{-6}\ mol/s} = 0.025$$

Under the given conditions, about 2.5% of the generated hydrogen can remain dissolved in the electrolyte. Therefore, at least 97.5% of the hydrogen must evolve as gas bubbles, consistent with the limited solubility of hydrogen in water.

Tables for the Supporting Information

Table S1. Reynolds number at the inlet tubes, calculated with the average velocity of the fluid.

Inlet tubes			
Q_{in} (mL/min)	Q_{in} (m^3/s)	u - average (m/s)	Re
10	1.67E-07	0.053	107
20	3.33E-07	0.106	214
30	5.00E-07	0.159	322
40	6.67E-07	0.212	429
50	8.33E-07	0.265	536
60	1.00E-06	0.318	643
70	1.17E-06	0.371	750
80	1.33E-06	0.424	857
90	1.50E-06	0.477	965
100	1.67E-06	0.531	1072
200	3.33E-06	1.061	2144

Table S2. Reynolds number at the inlet tubes, determined using the volume-averaged fluid velocity obtained from the 3D simulations.

Inlet tubes		
Q_{in} (mL/min)	u - volume average (m/s)	Re
10	0.027	54
20	0.054	108
30	0.081	163
40	0.107	217
50	0.134	271
60	0.161	325
70	0.188	380
80	0.215	434
90	0.242	488
100	0.268	542

Table S3. Reynolds number within one compartment (rectangular duct), determined using the volume-averaged fluid velocity obtained from the 3D simulations.

**Main cell**		
Q_{in} (mL/min)	u - volume average (m/s)	Re
10	0.002	19
20	0.006	56
30	0.010	98
40	0.014	133
50	0.017	165
60	0.020	194
70	0.023	222
80	0.025	248
90	0.028	271
100	0.030	294

Figures for the Supporting Information

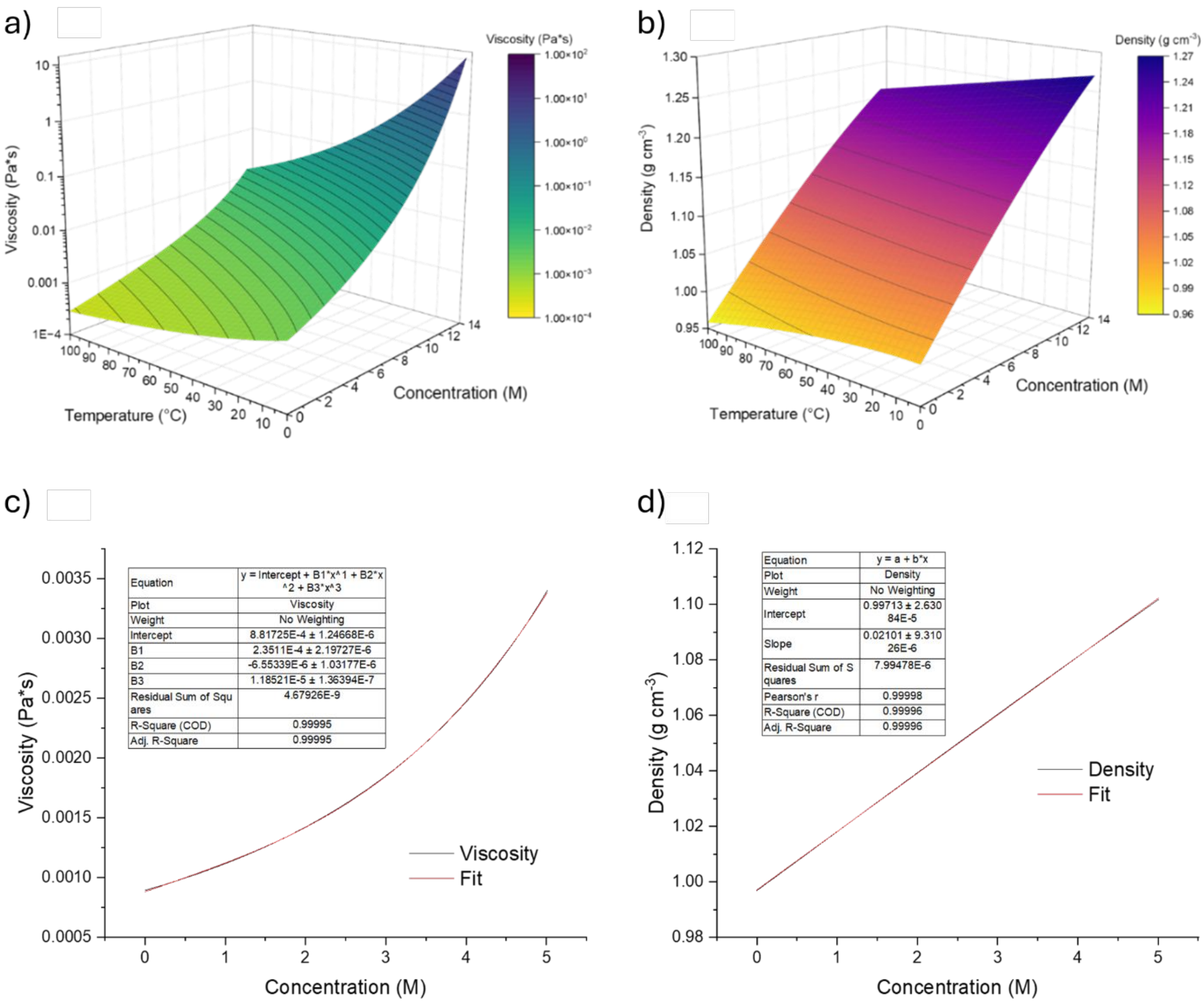

Figure S1: Water-glycerol solution properties as function of temperature and concentration: a) three-dimensional plot of viscosity from 0 to 14 M glycerol and 0-100 °C, b) three-dimensional plot of density from 0 to 14 M glycerol and 0-100 °C, c) viscosity from 0 to 5 M glycerol at 25 °C, d) density from 0 to 5 glycerol M at 25 °C.

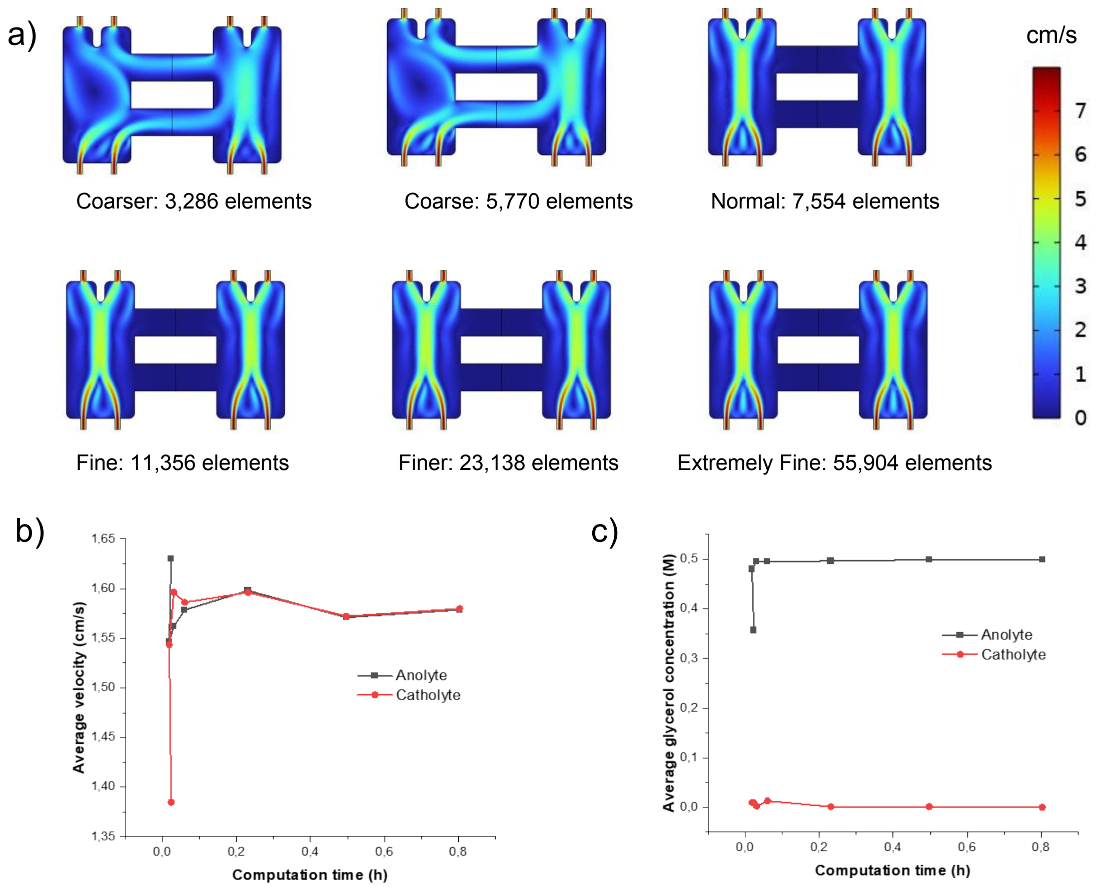

Figure S2. Mesh independence study for the two-dimensional (2D) geometry: a) velocity field maps illustrating effect of mesh size at an inlet flow rate of 25 mL/min using the density and dynamic viscosity of pure water, b) convergence of the average velocity at the catholyte and anolyte as a function of computation time. The computation time scales with the mesh size. c) converge study for the concentration of glycerol in the catholyte and anolyte as a function of computation time. Note that the liquid enters the compartments through the two lower inlets and exits through the top outlets, which are separated by the flow divider fin.

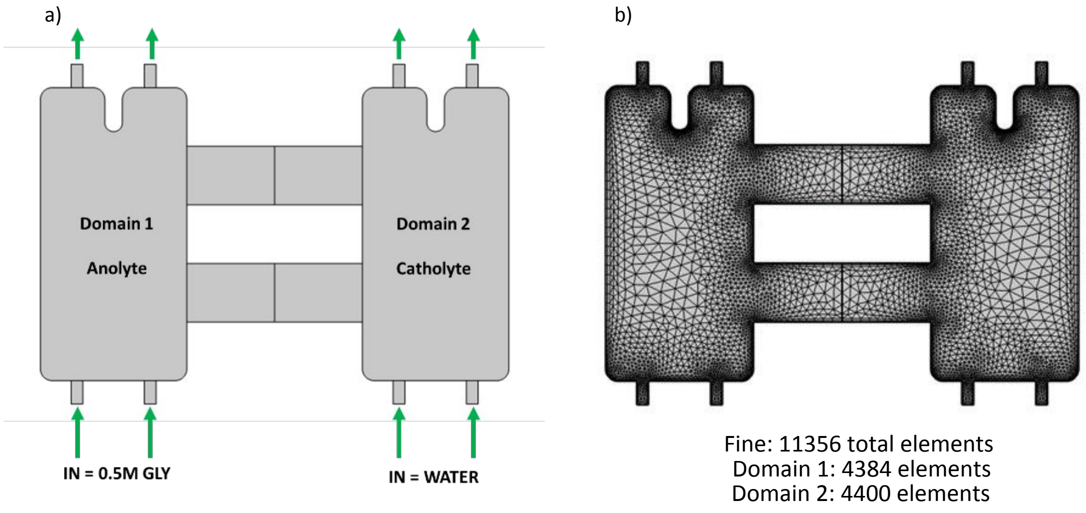

Figure S3: a) 2D inlet and outlet flow for PEC cell, including inlet concentrations, b) selected mesh for simulations.

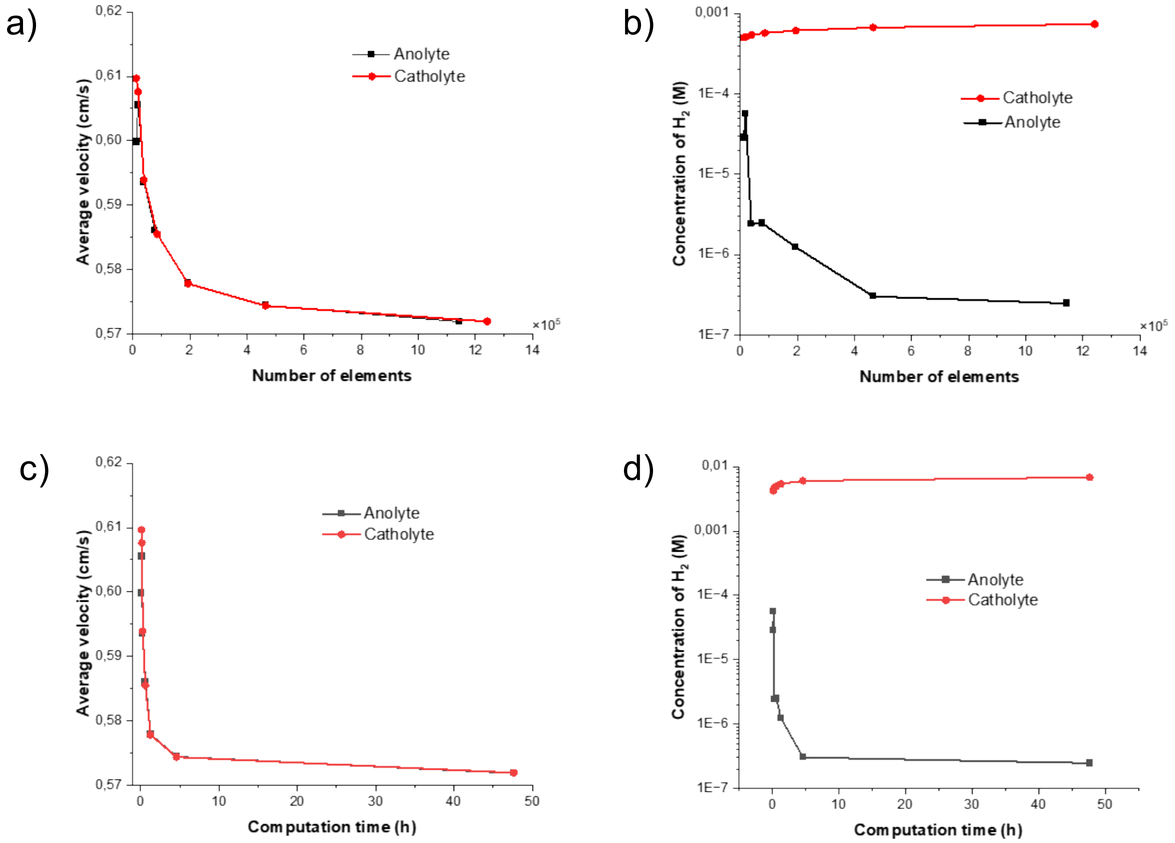

Figure S4. Mesh independence study for the three-dimensional (3D) geometry with two bridges: a) average velocity in the catholyte and anolyte as a function of mesh size, b) average hydrogen concentration in the catholyte and anolyte as a function of mesh size, c) average velocity in the catholyte and anolyte as a function of computation time, d) average hydrogen concentration in the catholyte and anolyte as a function of computation time. The selected "Fine" mesh consists of 1,165,144 elements.

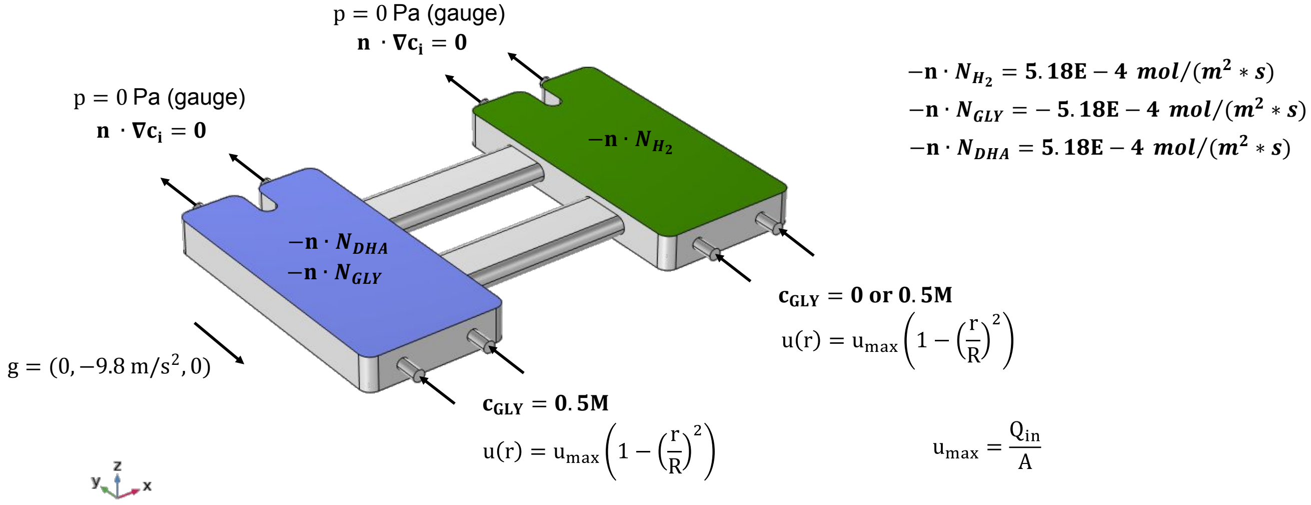

Figure S5. Boundary conditions implemented in the simulation. The text in bold corresponds to the transport of diluted species boundary conditions. The inlet consists of an inflow of 0.5 M glycerol for the anolyte and 0 or 0.5 M glycerol for the catholyte, set up as a concentration constraint. The blue upper surface is the anode where glycerol is consumed, and DHA is produced. The green upper surface is the cathode, where H_2 is generated. The molar flux from the electrode surface was determined using Faraday's law of electrolysis. For the CFD simulations the boundary conditions for the laminar flow are fully developed flow at the inlet and zero pressure at the outlet. For momentum transport, all the walls, except for the inlet and outlet, are no slip boundary conditions. In the case of mass transport, there is no flux through the walls, except for the inflow, outflow, and electrode surfaces.

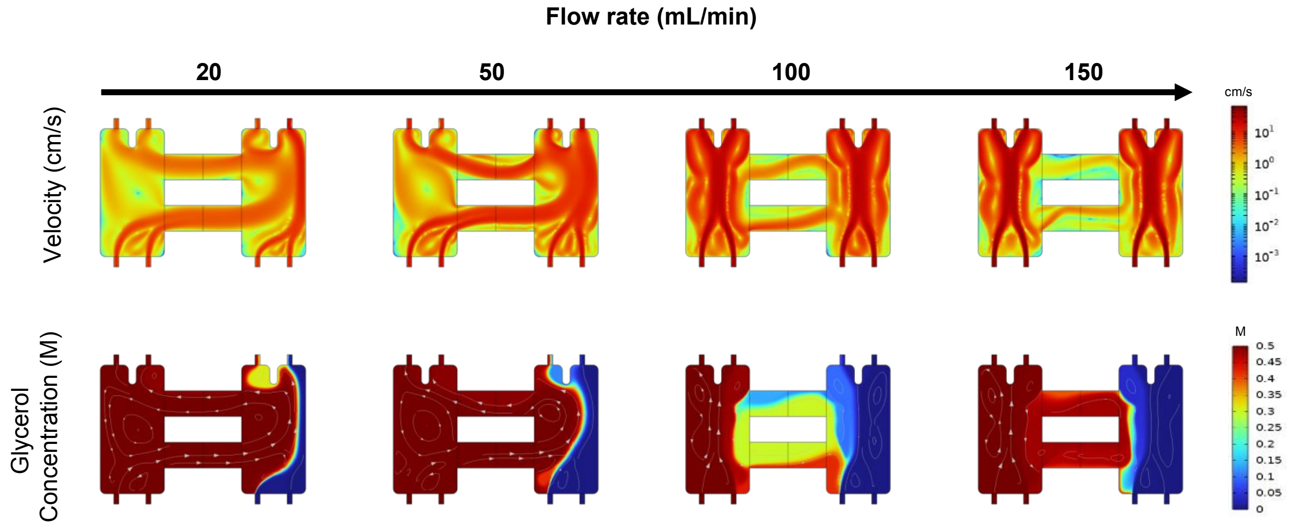

Figure S6. 2D fluid velocity and glycerol concentration at different flow rates. Density and viscosity depend on glycerol concentration. Note that the liquid enters the compartments through the two lower inlets and exits through the top outlets, which are separated by the flow divider fin.

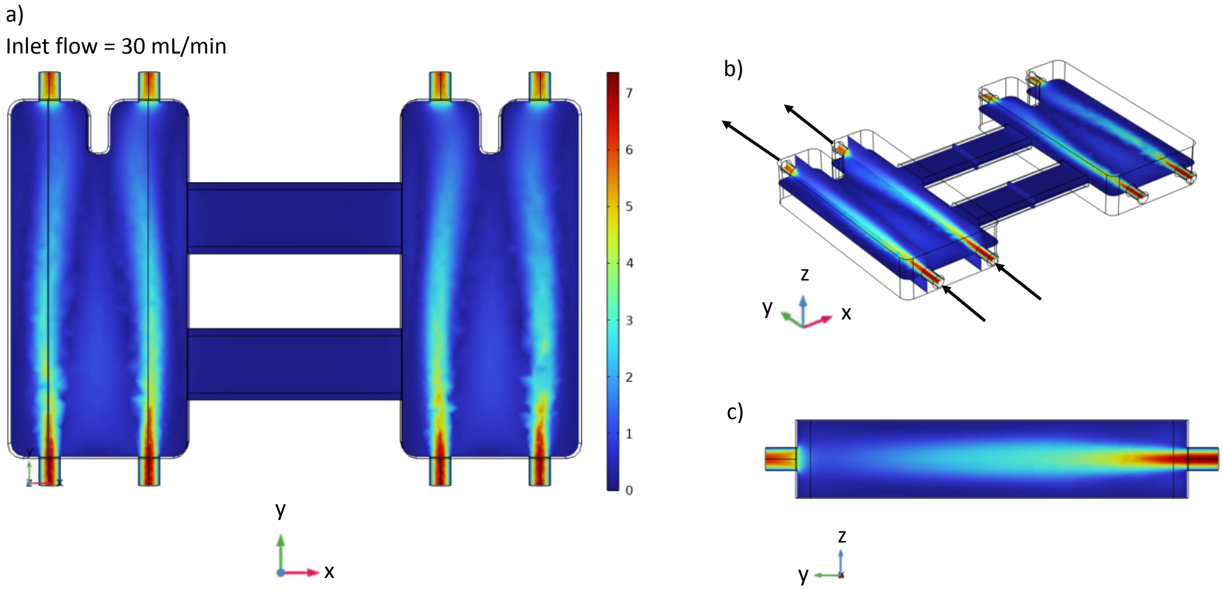

Figure S7. 3D fluid velocity profile, inlet flow rate of 30 mL/min. a) Front view (xy-plane), b) isometric view, c) side view (yz-plane). Note that the liquid enters the compartments through the two lower inlets and exits through the top outlets, which are separated by the flow divider fin.

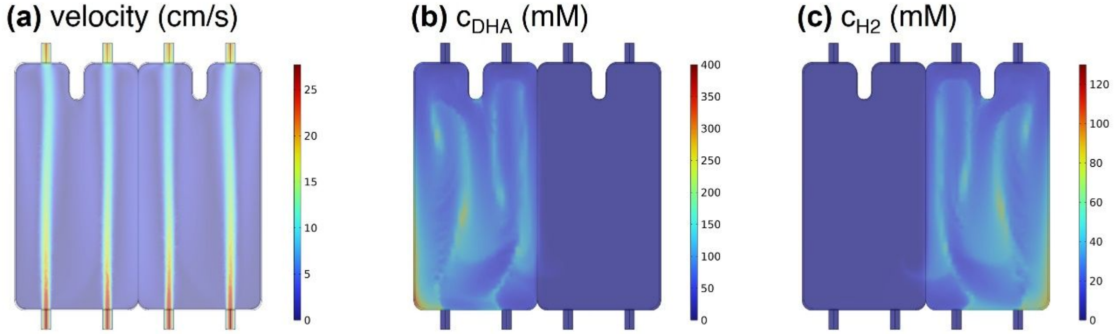

Figure S8. Three-dimensional simulation results for the device without a bridge connecting the two compartments. **(a)** Fluid velocity on the *xy*-plane (z = 3 mm), **(b)** DHA concentration (c_{DHA}) on the device surface, and **(c)** hydrogen concentration (c_{H2}) on the device surface, calculated at a flow rate of 50 mL/min. Note that the liquid enters the compartments through the two lower inlets and exits through the top outlets, which are separated by the flow divider fin.

References

(1) Favaro, M.; Kong, H.; Gottesman, R. In Situ and Operando Raman Spectroscopy of Semiconducting Photoelectrodes and Devices for Photoelectrochemistry. *J. Phys. Appl. Phys.* **2023**, *57* (10), 103002. https://doi.org/10.1088/1361-6463/ad10d3.

(2) Ralaiarisoa, M.; Krishnamurti, S. S.; Gu, W.; Ampelli, C.; Krol, R. van de; Abdi, F. F.; Favaro, M. In Situ Investigation of Ion Exchange Membranes Reveals That Ion Transfer in Hybrid Liquid/Gas Electrolyzers Is Mediated by Diffusion, Not Electromigration. *J. Mater. Chem. A* **2023**, *11* (25), 13570–13587. https://doi.org/10.1039/D3TA02050A.
